# MicroRNA-141-regulated KLK10 and TNFSF-15 gene expression in *hepatoblastoma* cells as a novel mechanism in liver carcinogenesis

**DOI:** 10.1038/s41598-024-63223-4

**Published:** 2024-06-12

**Authors:** Ahmed M. Awad, Emad Dabous, Mai Alalem, Nedaa Alalem, Mahmoud E. Nasr, Khaled A. Elawdan, Ghada M. Nasr, Walid Said, Kareem El Khashab, Mohamed S. Basiouny, Adel A. Guirgis, Hany Khalil

**Affiliations:** 1 Department of Molecular Biology, Genetic Engineering and Biotechnology Research Institute, University of Sadat City, 32897, Sadat City, Egypt; 2 Department of Molecular Diagnosis, Genetic Engineering and Biotechnology Research Institute, University of Sadat City, Sadat City, Egypt; 3 Molecular Diagnostics Department, Genetic Engineering and Biotechnology Research Institute, University of Sadat City, Sadat City, Egypt; 4 Microbiology and Chemistry Department, Faculty of Science, Benha University, Benha, Egypt; 5 Medical Laboratory Department, High Technology Institute of Applied Health Science, Badr Academy for Science and Technology, Badr City, Egypt; 6 School of Biotechnology, Badr University in Cairo, Badr City, Cairo Egypt

**Keywords:** Cancer, Cell biology, Genetics

## Abstract

Liver cancer is one of the most pivotal global health problems, leading hepatocellular carcinoma (HCC) with a significant increase in cases worldwide. The role of non-coding-RNA in cancer proliferation and carcinogenesis has attracted much attention in the last decade; however, microRNAs (miRNAs), as non-coding RNA, are considered master mediators in various cancer progressions. Yet the role of miR-141 as a modulator for specific cellular processes in liver cancer cell proliferation is still unclear. This study identified the role of miR-141 and its potential functions in liver carcinogenesis. The level of miR-141 in HepG2 and HuH7 cells was assessed using quantitative real-time PCR (qRT-PCR) and compared with its expression in normal hepatocytes. A new miR-141 construct has been performed in a CMV promoter vector tagged with GFP. Using microarray analysis, we identified the potentially regulated genes by miR-141 in transfected HepG2 cells. The protein profile of the kallikrein-related peptidase 10 (KLK10) and tumor necrosis factor TNFSF-15 was investigated in HepG2 cells transfected with either an inhibitor, antagonist miR-141, or miR-141 overexpression vector using immunoblotting and flow cytometry assay. Finally, ELISA assay has been used to monitor the produced inflammatory cytokines from transfected HepG2 cells. Our findings showed that** t**he expression of miR-141 significantly increased in HepG2 and HuH7 cells compared to the normal hepatocytes. Transfection of HepG2 cells with an inhibitor, antagonist miR-141, showed a significant reduction of HepG2 cell viability, unlike the transfection of miR-141 overexpression vector. The microarray data of HepG2 cells overexpressed miR-141 provided a hundred downregulated genes, including KLK10 and TNFSF-15. Furthermore, the expression profile of KLK10 and TNFSF-15 markedly depleted in HepG2 cells transfected with miR-141 overexpression accompanied by a decreasing level of interleukin 6 (IL-6) and tumor necrosis factor-alpha (TNF-α), indicating the role of miR-141 in HepG2 cell proliferation and programmed cell death. Interestingly, the experimental rats with liver cancer induced by Diethylnitrosamine injection further confirmed the upregulation of miR-141 level, IL-10, and TNF-α and the disturbance in KLK10 and TNFSF-15 gene expression compared with their expression in normal rats. The in-silico online tools, IntaRNA and *miRWalk were* used to confirm the direct interaction and potential binding sites between miR-141 and identified genes. Thus, the seeding regions of potential targeted sequences was cloned upstream of luciferase reporter gene in pGL3 control vector. Interestingly, the luciferase activities of constructed vectors were significantly decreased in HepG2 cells pre-transfected with miR-141 overexpression vector, while increasing in cells pre-transfected with miR-141 specific inhibitor. In summary, these data suggest the crucial role of miR-141 in liver cancer development via targeting KLK10 and TNFSF-15 and provide miR-141 as an attractive candidate in liver cancer treatment and protection.

## Introduction

Typically, miRNAs are a class of non-coding RNAs that are between 22 and 24 nucleotides long. These single-stranded RNA molecules control gene expression post-transcriptionally in two separate ways (near-perfect complementation and partial complementation). Notably, because one miRNA species may target one or more messenger RNAs (mRNAs), which can be targeted by several miRNAs with numerous binding sites in their sequence, miRNAs may govern important cellular functions. Expression of one or more transcripts can be negatively regulated by base-pairing of miRNAs with specific mRNAs. Additionally, miRNAs control the expression of 30% of human genes, many of which are linked to tumors or are found in unstable sections of the genome, confirming their critical role in the development of human cancer^1^. The targeted mRNA is cleaved and degraded, or the protein translation is inhibited relying on the complementary degree between miRNA and mRNA^2^. Each miRNA negatively regulates target genes either through binding to the 3′ untranslated region (UTR) or to the open reading frame of mRNAs which are both involved in RNA interference mechanism. In vertebrates, mRNA transcripts are usually cleaved by a miRNA-associated complex, known as RNA-induced silencing complex (RISC)^3^. Importantly, miRNAs are key players in many cancer types, such as colorectal cancer and pancreatic cancer via the interaction between them and many targeted genes required for cancer development of these types of cancer. For instance, miR-217 can reduce the expression of mutant protein, Kirsten RAS (KRAS), in pancreatic cancer cells^4,5^. In addition, in pancreatic cancer, the KRAS pathway was targeted by miR-96, which acts as a tumor suppressor factor. Interestingly, miRNA 141 modulates the expression of tumor suppressor-deleted liver cancer 1(DLC-1) which is a Rho-GTPase-activating protein in liver cancer^6^. It was reported that DLC-1 was targeted by miR-141 and increase in miR-141 leads to increase in HCV infection and DLC-1 deletion and HCC severity^7^. It is well known that miR-141 is a member of the miR-200 family which is involved in many cell activities including tumorigenesis and tumor cell survival. The binding between the miR-200 family and RAS supper family 22 (RASSF2) verified using many techniques like dual-luciferase reporter assay and northern blotting^8,9^. Additionally, this was confirmed by direct binding interaction between the miR-200 family and RASSF2^9^. Notably, the kallikrein-related peptidase 10 (KLK 10) factor plays a pivotal role in tumorigenesis in both breast cancer and prostate cancer^10^. In addition, the KLK 10 overexpression in prostate cancer (PC) specifically in PC-3 cell line leads to decelerate tumor proliferation^11^. Additionally, high serum levels of KLK10 were reported to be significantly associated with late-stage high-grade serous ovarian tumors^12^.

Consequently, the expression of KLK10 mRNA was completely absent in more than half of the ductal carcinomas and 29 of 30 of the infiltrating ductal carcinomas according to in situ hybridization on tissue sections from normal breast and in infiltrating ductal carcinoma, while KLK10 expression was investigated in normal and hyperplasia samples^13^. Therefore, the loss of KLK10 expression is required for tumor progression. The expression of KLK10 in breast cancer also underlies a unique mechanism of regulation for the gene, with downregulation in different cancer types like prostate and testicular cancers and leukemia^13^. In the same context, the tumor necrosis factor superfamily (TNFSF) is a collection of cytokines that share structural similarities and exhibits a diverse array of functions in the regulation of both immunity and inflammation. The differentiation, proliferation, and programmed death of many different types of cells^14^. Tumor necrosis factor superfamily-15 (TNFSF-15) like other TNFSF members is multifunctional. TNFSF-15 is predominantly produced by vascular endothelial cells and specifically inhibits endothelial cell growth. TNFSF-15 is also a T-cell co-activator and a stimulator of dendritic cell maturation which is considered an important step in host immune system activation^15,16^. TNFSF-15 thus plays a key role in the maintenance of vascular and immune system homeostasis. TNFSF-15 diminishes in ovarian cancer as the disease progresses while in prostate cancer, the 5′ adenosine monophosphate-activated protein kinase (AMPK) () enhances transcription levels of TNFSF-15 and inhibits tumor growth. TNFSF-15 expression is also modulated by interlukines-1 beta (IL-1β) and chondroitin sulfate in osteoarthritis patients^16^. Hence, there is ongoing interest in observing the role of TNF as a mediator in HCC as well.

Additionally, Interleukins (ILs) are cytokines that mediate leukocyte crosstalk and modulate proliferation, differentiation, growth, survival, and functions of immune cells. Cytokines such as IL-6, IL-8, and IL-10 are produced and secreted by activated immune cells such as macrophages, monocytes, lymphocytes, and many cancer cell types. Such cytokines exploit in either an autocrine or paracrine manner which promote cancer cell infiltration, metastasis, and acute phase responses in many cancers. In addition to their role in immunity, IL-6, IL-8, and IL-10 promote growth, invasion, metastasis, and drug resistance in various tumor types. Low serum IL-6 levels enhance immune responses and inhibit the growth of cancer cells, whereas high serum IL-6 levels decrease immunity and improve infiltration by tumor cells. Elevated serum IL-6 levels correlate with tumor stage and poor survival^17^. In the same context, the IL-6 elevation is deregulated in cancer besides its abundance in the tumor microenvironment; therefore, its occurrence is a potent correlation between cancer and inflammation^18,19^. The inhibition of IL-6 could be considered a potential therapeutic strategy for the treatment of cancers via IL-6-dominated signaling^19^. Indeed, the role of miR-141 role in liver cancer is still an attractive area of research. In this study, we aim to further investigate the molecular function of miR-141 in liver cancer cell lines to identify its potential targeted genes that may contribute in liver cancer treatment and protection.

## Materials and methods

### Cell lines and cell culture

In this study, HepG2 and HuH7 cell lines and the normal hepatocytes were obtained from (VACSERA, Giza, Egypt) and regularly checked for mycoplasma contamination. Cell lines and normal cells were cultured in Roswell Park Memorial Institute (RPMI) 1640 media, containing 25 mM HEPS, 4 mM l-glutamine, and 10% of heat-inactivated bovine serum albumin (BSA) and incubated in CO_2_ incubator (LabTech, DAIHAN LABTECH CO., LTD.) at 37 °C and relative humidity of 95%^20^.

### Experimental animals

Ten male albino rats were obtained from VACSERA (Cairo, Egypt), weighing 100–120 g. The rats were housed in the animal house for a week under the optimal conditions of humidity (40–70%) and temperature (27–30 °C) and were fed with a standard pellet diet and water. The HCC model was induced by an intraperitoneal administration of Diethylnitrosamine (DEN) (CAS 55-18-5, Sigma, UAS) (200 mg/kg). The severity of HCC was increased by the injection with carbon tetrachloride (CCL4) (CAS 56-23-5, Sigma, USA). To confirm the induction of HCC, randomly selected rats were sacrificed and the activities of liver enzymes and other biological indicators were evaluated in a schedule (1 and 2 weeks upon induction). For the histological analysis of rats with HCC at the same schedules, the liver samples were fixed in 10% neutral buffer formalin for 24 h. Afterward, samples were processed routinely and then embedded in paraffin wax. Blocks were prepared, and 5 ml thick sections were performed using a microtome. Liver sections were deparaffinized using xylene and ethanol. The slides were treated through the classic method before being stained with hematoxylin and eosin (H&E)^21^. Later, all slides were examined via the inverted microscope. Furthermore, the relative gene expression of KLK10 and TNFSF-15 parallel with the expression level of miR-141 was monitored in liver samples of rats with HCC that normalized to samples derived from control rats. The concentration of TNF-α and IL-10 in the blood serum of experimental rats was achieved using mouse ELISA kits (Abcam 100747 and Abcam 100697, respectively).

### Cloning and cloning strategy

To perform the miR-141-over-expression vector, GFP plasmid with CMV promoter was used to clone the full-length miR-141 between the CMV promoter and GFP sequence. To generate the construct pCMV-miR-141-GFP, the full-length miR-141 was isolated from HepG2 cells using the following specific oligonucleotides containing restriction sites specific for Sac1 and Pst1: Sac1-For-5ʹ-gagctccgctaacactgtctggtaaag-3ʹ and Pst1-Rev-5ʹ-5ʹ-ctgcaggtgcagggtccgaggt-3ʹ. Accordingly, total RNA was extracted from cultured HepG2 cells using TRIzol (Invitrogen, USA). The extracted total RNA was then purified using the RNeasy Mini Kit (Qiagen, USA), followed by the purification of small miRNAs using the RNeasy MinElute Cleanup Kit (Qiagen, USA) according to the manufacturer’s protocol. cRNA was synthesized from total RNA using miR-141-Pst1-Reverse primer and the following reagents: 4 µL 10× RT buffer (Promega), 4 µL RNA (1 µg/µL), 4 µL dNTPs (25 mM), 0.5 µL RNase inhibitor (5 U/µL) (Promega) and 0.5 µL M-MuLV reverse transcriptase (100 U/µL) (Promega) up to 20 µL final volume using RNase free water. The mixture was incubated at 42 °C for 3 h then followed by 10 min at 95 °C to stop the reaction. The synthesized cRNA was then used to amplify the full-length miR-141 using conventional PCR by the indicated miR-141 specific primers. PCR product was then loaded in agarose gel 1%, eluted and digested with Sac1 and Pst1 as well as pCMV-GFP plasmid as recommended in NEB conditions. The open vector was loaded in agarose gele, which then eluted followed by overnight incubation at 4 °C with miR-141 digested fragments and 5U of T4 DNA ligation, 4 µL 5× ligation buffer (Promega), 2 µL from cRNA (100 mM), 2 µL from the vector (1 µM) adjested to a final volume of 20 µL. Further, the individual coding sequences (almost 1000 bp) of KLK10 and TNFSF-15 by which miR-141 could interact, were cloned upstream of luciferase reporter gene in the pGL3-control vector with SV40 promoter (Promega, Germany). First, we amplified the specific fragment from genomic DNA of cultured HepG2 cells using Platinum SuperFi DNA polymerase (Invitrogen, Germany) based on the predicted seeding region of miR-141 and targeted mRNA using the following primer sequences;

- KLK-10-(1000)-F-(5ʹ-TTGTCTGCACTGTTCAAACCTCTG-3ʹ) and
- KLK-10-(2000)-R-(5ʹ-TGAGCCACCACACCCAGCGGACAC-3ʹ),
- TNFSH-15-(115)-F-(5ʹ-AGAGGTGCCT CCAGGAGCAG CAGG-3ʹ) and
- TNFSH-15-(1100)-R-(5ʹ-TTCCAGTTAGTACTCTCATCAGTAAG-3ʹ).

The pGL3-control vector was digested with HindIII using FastDigest HindIII (Thermo Scientific, USA). The digested vector was loaded and electrophoresed in 1% agarose gel and was then eluted from the gel using a QIAquick Gel Extraction kit (Qiagen, USA). The blunt-end protocol was used to insert the amplified fragment in the pGL3 open vector using a TOPO-Blunt-End Cloning kit (Invitrogen, Germany). Briefly, the amplified fragment was incubated with the pGL3 opened vector in a concentration ratio of 3:1 and five units of ExpressLink T4 DNA ligase (Invitrogen, Germany) for 30 min at room temperature. The miR-141 overexpression vector and luciferase reporter constrcats were transformed into competent *E.cloi* strain by heat shock (42 °C for 45 s). Transformed *E.coli* was grown in a Petri dish with agar media containing ampicillin at 37 °C for 3 days. A selected single colony of *E.coli* was grown in broth media at 37 °C overnight. Maxi-Prep kit (Qiagen, USA) was used to amplify the constructs, which were then checked for the correct insert and proper orientation using the restriction sites map of the new construct prepared by the cloning manager program.

### Transfection protocol

The normal hepatocytes and HepG2 cells were grown on a complete RPMI medium and were overnight cultured in 6-well plates with confluency of about 80%. Cells were then transfected with 1.25 µg/ml pCMV-GFP-miR-141 construct using Lipofectamine LTX (Invitrogen, USA), according to the manufacturer’s protocol. Transfected cells were incubated for 2 days, followed by total RNA isolation for qRT-PCR, staining for flow cytometry, or protein purification for immunoblotting assay. Other cells seeded in six-well plates with confluency of about 70% were transfected with a respective inhibitor antagonist, miR-141 (5′-ACAACCACTGTCTGGTAAAG-3′). The cells were transfected with 1.25 µg of inhibitor/ml using 20 µL Lipofectamine LTX due to the manufactures instructions (Invitrogen, USA), prepared in 500 µL optimum media. Cells transfected with the same concentration of transfection reagents were severed as control-transfected cells. Transfected cells were incubated for 2 days. The knockdown efficiency of miR-141 and the relative expression of indicated genes were monitored in transfected cells using qRT-PCR. Flow cytometry assays for KLK10 and TNFSF-15 protein were assessed on day two post-transfection^22^.

### Transfection cytotoxicity and proliferation assay

Transfected cells' cytotoxicity and cell viability were monitored to achieve the anti-proliferation properties of miR-141 overexpressing vector. Accordingly, the normal hepatocytes and HepG2 cells were seeded in a 6-well plate in triplicates and were transfected with miR-141 overexpressing vector or specific inhibitor as previously described. Forty-eight hours post-transfection, cell morphology and number of living cells were monitored using an inverted microscope and hemocytometer, respectively^23,24^. To investigate cell viability upon transfection, the normal cells and HepG2 cells were cultured in triplicate in 96-well plates with a density of 10 × 10^3^ cells per well, followed by transfection with different concentrations of miR-141 overexpressing vector or specific inhibitor (1.25–20 µg/ml). Other cells treated with the same concentration of transfection reagents served as control-transfected cells. Cell viability rate was achieved using an MTT colorimetric assay kit (Sigma-Aldrich, Germany). Briefly, transfected cells were washed using phosphate buffer saline (PBS), and 100 µL complete media was added to each well. 10 µL MTT solution was added to each well with a gentile piptting. The plate was then incubated for 1 h at 37 °C. Finally, 100 µL SDS-HCl solution was added to each well in the plate was and incubated for 4 h at 37 °C. Then cell viability was determined due to the converted water-soluble MTT to an insoluble formazan which was determined by optical density at 570 nm. For cotransfection with luciferase reporter constructs, HepG2 cells were cultured in black 96/well plates with a 10 × 10^3^ cell/well density and incubated overnight. Then, the cells were transfected with either miR-141 overexpression vector or miR-141 specific inhibitor using 125 ng from each, 5µL Lipofectamine LTX, and 20 µL optimum media to transfect each well. Two days later, the transfected cells were cotransfected with 125 ng of pGL3 constructs using 5 µL Lipofectamine and 20 µL optimum media per well. The cells were then incubated for 24 h and prepared for luciferase dual assy.

### Annexin-V assay

Early and late apoptosis determination was performed using an annexin-V (FITC)/propidium iodide (PI) assay kit (BD Biosciences) according to the manufacturer's protocol. Briefly, 10 × 10^4^ HepG2 cells were cultured in a 25 ml cell culture flask and incubated overnight under the same described conditions for cell culture. The cells were transfected with 1.25 µg/ml of either miR-141 overexpression vector or specific inhibitor and incubated for 24 h. Transfected cells were then collected and washed twice with PBS, and were resuspended in the kit's Binding Buffer. Then, 100 μl of the cell suspension was incubated with 5 μl of Annexin‐V conjugated fluorescein isothiocyanate (FITC) and 5 μl of PI for 15 min in the dark at room temperature. Then 500 μl of the binding buffer was appended, and the cells were analyzed by flow cytometry within one hour.

### Microarray analysis

Total RNA was purified from transfected cells using TRIzol and RNA preparation method (Invitrogen, USA) using glycogen as a carrier following the manufacturer’s protocol with a few modifications. Xylene and ethanol treatment in the precipitation step were excluded. To reduce RNA degradation, the incubation steps at 55 °C and 80 °C were shortened to 12 instead of 15 min. RNA elution was performed by RNase free water from the column, quantified by NanoDrop, and stored at − 80 °C. RNA quality was confirmed by an Agilent 2100 bioanalyzer (Agilent Technologies) and a NanoDrop 1000 spectrophotometer. In brief, 600 ng of total RNA were reverse transcribed and amplified using an oligo-dT-T7-promotor primer, and the resulting cDNA was labeled either with Cyanine 3-CTP or Cyanine 5-CTP. The microarray experiments were scanned using a DNA microarray laser scanner (DNA Microarray Scanner BA, Agilent Technologies) at 5 µm resolution using Ambion ship and Exiqon ship according to the manufacturer’s instructions. The original microarray images were analyzed with the Image Analysis/Feature Extraction software G2567AA (Version A.7.5, Agilent Technologies) using default or standard miRNA microarray settings. Non-uniformity outlier flagging was performed with a 5 × default value of the constant coefficient of variation for features ((CV)2 Term (A) = 0.55) and background ((CV)2 Term (A) = 0.45). Intensities were normalized by local background correction and rank consistency filtering with LOWESS normalization. The intensity ratios were calculated using the most conservative estimate of error between the Universal Error Model and propagated error^25^. A Two-fold change expression cut-off for ratio experiments was applied with anti-correlation of color-swapped ratio profiles, depicting the microarray analysis as highly significant (*P *value < 0.01), robust, and reproducible.

### Monitoring miR-141 expression level

To detect the expression level of miR-141, total RNA extraction was conducted from cultured cell lines and then transfected HepG2 cells (48 h post transfection) using TRIzol (Invitrogen, USA). The extracted total RNA was then purified using the RNeasy Mini Kit (Qiagen, USA), followed by the purification of small miRNAs using RNeasy MinElute Cleanup Kit (Qiagen, USA) according to the manufacturer’s protocol. The relative expression of miR-141 was detected by RT-PCR in two different steps. First, cDNA was performed from small miRNAs by using reverse transcriptase reaction followed by the amplification step using miR-141 and RNU6B specific TaqMan microRNA assays (Applied Biosystem, Darmstadt, Germany), according to the manufacturers protocol. Levels of RNU6B were used for normalization. To perform cDNA from small miR-141, the following reagents were prepared for reaction: 0.15 µL dNTPs (100 mM), 1 µL reverse transcriptase (50 U/µL), 1.5 µL 10× reverse transcriptase buffer, 0.2 µL RNase inhibitor (20 U/µL), 5 µL purified miRNAs (10 ng/µL) and 1 µL from each primer up to final volume of 20 µL using RNase free water. The mixture then incubated at 42 °C for 30 min, and then followed by 5 min at 85 °C for the enzyme inactivation. The resulting cDNA then was used as a template to amplify both miR-141 and RNU6B by using the following parameters in quantitative real-time PCR (qRT-PCR) machine: 95 °C for 5 min, 40 cycles (95 °C for 15 s, 60 °C for 15 s and 72 °C for 15 s) and 72 °C for 3 min^5^. The same procedure was applied to detect the relative expression level of miR-141 in the liver tissue of experimental rats.

### Gene expression profiling

To detect the relative gene expression, qRT-PCR was used to perform cDNA construction and amplification in one step using the purified total RNA as a template. Total RNA from transfected HepG2 cells was extracted 48 h post-transfection using TRIzol and purified using the RNeasy Mini Kit (Qiagen, USA). The relative expression of klk10, TNF-SF15 was detected using the QuantiTect SYBR Green PCR Kit (Qiagen, USA) in miR-141 transduced HepG2 cells or miR-141 depleted cells. The oligonucleotides listed in Table 1 have been used as specific primers for indicated genes. Level of amplified GAPDH was used for normalization. The following reagents were prepared for each reaction; 10µL SYBR green, 0.2 µL RNase inhibitor (20 U/µL), 0.25µL reverse transcriptase (50 U/µL), 1µL purified total RNA (100 ng/µL) and 0.5 µL from each primer up to a final volume of 20 µL using RNase free water. According to the manufacturer’s protocol, the following PCR parameters were sued to construct and amplify cDNA, in one step, from a total RNA template: 50 °C for 30 min, 95 °C for 3 min, 40 cycles (95 °C for 30 s, 60 °C for 15 s, 72 °C for 30 s) and 72 °C for 10 min^26^.

**Table 1:** Oligonucleotides sequences to quantify miR-141 and mRNA of indicated genes.

Description	Primer sequences 5ʹ–3ʹ
MiR-141-sense	CGCTAACACTGTCTGGTAAAG
MiR-141 antisense	GTGCAGGGTCCGAGGT
MiR-U6-sense	GCTTCGGCAGCACATATACTAAAAT
MiR-U6-antisense	CGCTTCACGAATTTGCGTGTCAT
KLK 10-sense	GCCCGATCCCAAACTCCATT
KLK 10-antisense	GGGTAAACACCCCACGAGAG
TNFSF-15-sense	AAGGTTTACTGCCACCTCCAGAA
TNFSF-15-antisense	TTACCAATGTAATAACAACTGTT
GAPDH-sense	TGGCATTGTGGAAGGGCTCA
GAPDH-antisense	TGGATGCAGGGATGATGTTCT

### Immunoflorescent assay

HepG2 cells were cultured over coverslips in 24-well plates with a density of 50 × 10^3^ cells/well and were incubated overnight. The cells were transfected with 12.5 ng of CMV-GFP constructs using 10 µL Lipofectamine LTX and incubated for 2 days. Transfected cells were then fixed by adding 500 µL cold methanol for 3 min at RT. The permeabilization was performed by adding 500 µL phosphate-buffered saline (PBS) with 0.01 Triton-X-100. After washing, the cells were stained with 1 μg/ml of the DNA fluorescent dye (DAPI) for 15 min at RT. Coverslips were placed onto fluorescent slides using mowiol and samples were examined by a Leica TCS-SP laser scanning confocal microscope. Photomicrographs were processed using Adobe Photoshop 7.0M (Adobe Systems) and Microsoft PowerPoint^27^.

### Flow cytometry analysis

Flow cytometry was used to assess the kinetic expression of KLK10, and TNFSF-15 in transfected HepG2 cells. Accordingly, the transfected cells were washed twice with PBS and then were trypsinized for 3 min. The complete RPMI medium was added to the trypsinized cells, and then the cells were centrifuged for 5 min at 3000 rpm. Afterwards, the supernatant was discarded and pellet was resuspended and conducted in PBS for washing and incubated for 3 min in cold methanol for fixation. The cells were resuspended in PBS with Triton-X-100 (0.1%) and incubated for 3 min for permeabilization. For primary antibody staining, the cells were resuspended and followed by overnight incubation at 4 °C in the PBS supplemented with 1% BSA and the diluted mouse monoclonal anti-KLK10 (1–500) (Sino Biological, China). After washing with pure PBS, the cells were centrifuged and resuspended in the PBS with 1% BSA and 1–1000 secondary antibodies (goat anti-mouse IgG, Alexa Fluor 488, Abcam, USA). The cells were then incubated in dark conditions for 2 h. The same instructions was followed for staining the cells with the primary and secondary antibodies against TNFSF-15 using rabbit monoclonal anti- TNFSF-15 (1–500) (Ab 275022, Abcam, USA) and goat anti-rabbit IgG (1–1000) (Alexa Fluor 594, Abcam, USA)^28^. Finally, the flow cytometry assay (BD Accuri 6 Plus) was used to assess the protein levels using a resuspended pellet in 500 µL PBS^29^.

### Western blot analysis

The expression profile of KLK10 and TNFSF-15 proteins were double-checked using an immunoblotting assay. Total protein was extracted from transfected and infected HepG2 cells using the complete lysis and extraction buffer, RIPA (ThermoFisher, USA). Following this step, the protein was denaturized via a loading buffer containing 10% sodium dodecyl sulfate (SDS) followed by boiling at 95–100 °C for 5 min. 100 ng of denaturized protein was loaded in 10% sodium dodecyl sulfate–polyacrylamide gel. The electrophoresis was carried out for 4 h at 75 V using the Bio-Rad Mini-Protean II electrophoresis unit. The protein bands were transferred onto nitrocellulose membranes (Millipore, MA, USA) using Bio-Rad electro-blotting system (Bio-Rad Mini Trans-Blot Electrophoretic Transfer Cell), the membrane was incubated for one hour at room temperature in 30 ml of Tris Buffered Saline containing 5% non-fat dry milk and 0.1% Tween-20 (pH 7.5). Subsequently, the membrane was incubated overnight at 4 °C individually with the previously described primary antibodies targeted KLK10 and TNFSF-15. The membrane was then washed twice using WesternBreeze solution 16x (Invitrogen, USA) followed by 2 h incubation at room temperature with mouse monoclonal anti-β-actin (Sigma, Hamburg, Germany). At the end, all membranes were washed twice and incubated at RT for 2 h with either anti-mouse or anti-rabbit ready-to-use 2° Solution Alkaline-Phosphates (AP) Conjugated (Invitrogen, USA). The chromogenic detection of expected bands was performed immediately using AP substrate (WesternBreeze, Invitrogen, USA)^30,31^. All stained membranes were scanned by using a gel documentation system.

### Enzyme-linked immunosorbent assay (ELISA)

For the quantification of the released IL-4, IL-6, IL-10 and TNF-α, ELISA assay was performed using human ELISA kits (Abcam 215089, Abcam 46027, Abcam 46034, and Abcam 46087, respectively). Consequently, HepG2 cells were overnight cultured in a 96-well plate with a density of 10,000 cells/well. Afterwards, the cells were transfected using a 20 µL optimum medium which contains 25 µg/well of either miR-141 inhibitor or precursor suspended in 2 µL HyperFect. Transfected cells were then incubated for 6 h; then, a fresh RPMI medium was added to each well instead of the transfection medium, and cells were incubated for 2 days. Finally, both treated and transfected cells were incubated for different periods ranging from 0, 6, 12, 24, and 48 h. At each time, 50 µL of the lysed cells were transferred into the ELISA plate and incubated for 3 h at RT with the volume similar to the control solution and 1X biotinylated antibody. Following washing, 100 µL of 1× streptavidin-HRP solution was added to each well which then was incubated for 30 min in the dark. Then, 100 µL of the chromogen TMB substrate solution was added to each well, followed by 15 min incubation at RT away from the light. The stop solution was added, and the absorbance of each well was monitored using 450 nm^29,32^.

### Prediction tools and bioinformatics analysis

The Freiburg RNA online tool: IntaRNA program was used to predict the possible interactions between miR-141 and targeted genes sequence. The miR-141 sequence was obtained from mirbase website, while individual gene sequence has been obtained from National Library of Medicine (https://www.ncbi.nlm.nih.gov). In-silico online tool, *miRWalk*, was also used to validate and confirm the direct interaction between miR-141 and identified targeted genes using the following link http://mirwalk.umm.uni-heidelberg.de. This analysis is based on the following criteria: miRNA seed region, conserved site, free energy, and site accessibility. For the evaluation of the overlapping between the targeted genes for miR-141, the protein–protein interaction was performed for identified proteins. For this aim, we used the Search Tool for the Retrieval of Interacting Genes (STRING) version 12 database (https://string-db.org/). The STRING database involves big data for proteins' physical, functional, and predicted interactions between different proteins. The STRING databases can also create a model of a network on interactions between our selected proteins. Besides, STRING can create a model for the pathways for the selected proteins and their biological processes via linking to the Kyoto Encyclopedia of Genes and Genomes (KEGG) and a Gene Ontology (GO) database^33^.

### Data analysis

The average absorbance values in four replicates of transfected cells and related standard deviation (STD) of MTT assay and LDH detection kit were performed by Exile and represented three independent experiments. The mean average of living cells upon transfection and the STD were calculated using Exile formulation as a mean value of three replicates represented from two independent experiments. Likewise, the mean concentration of produced interleukins in the time course experiment has been calculated and performed in two independent experiments with three replicates for each using an Exile sheet. The quantification analysis of mRNA from the qRT-PCR assay was conducted using the Delta-Delta Ct method, which involved the following equations: (1) delta-Ct = Ct value for the target gene − Ct value for GAPDH, (2) delta-delta Ct = delta Ct value for experimental samples − delta Ct value for control samples, and (3) quantification fold change = 2^(−delta-delta Ct)^^30,34,35^. Exile sheets were used to prepare all diagrams, pikes, and histograms of represented data. Statistical analysis was performed using the student's two-tailed t-test, and a p-value ≤ 0.05 was considered statistically significant.

### Ethics approval and consent to participate

The current work is ethically approved by the Ethical Committee of the University of Sadat City. All animal-handling actions as well as samples gathering and discarding protocol were performed in accordance with ARRIVE guidelines, including anesthesia and euthanasia methods at the Institutional Animal Care and Use Committee (IACUC) with oversight of the faculty of Veterinary Medicine, University of Sadat City, Sadat City, Egypt (Ethical approval number: VUSC-025-1-22). The studies were conducted in accordance with the local legislation and institutional requirements.

## Results

### Preparing the miR-141 overexpression system based on miR-141 expression in liver *cancer* cell lines

**Figure 1:**
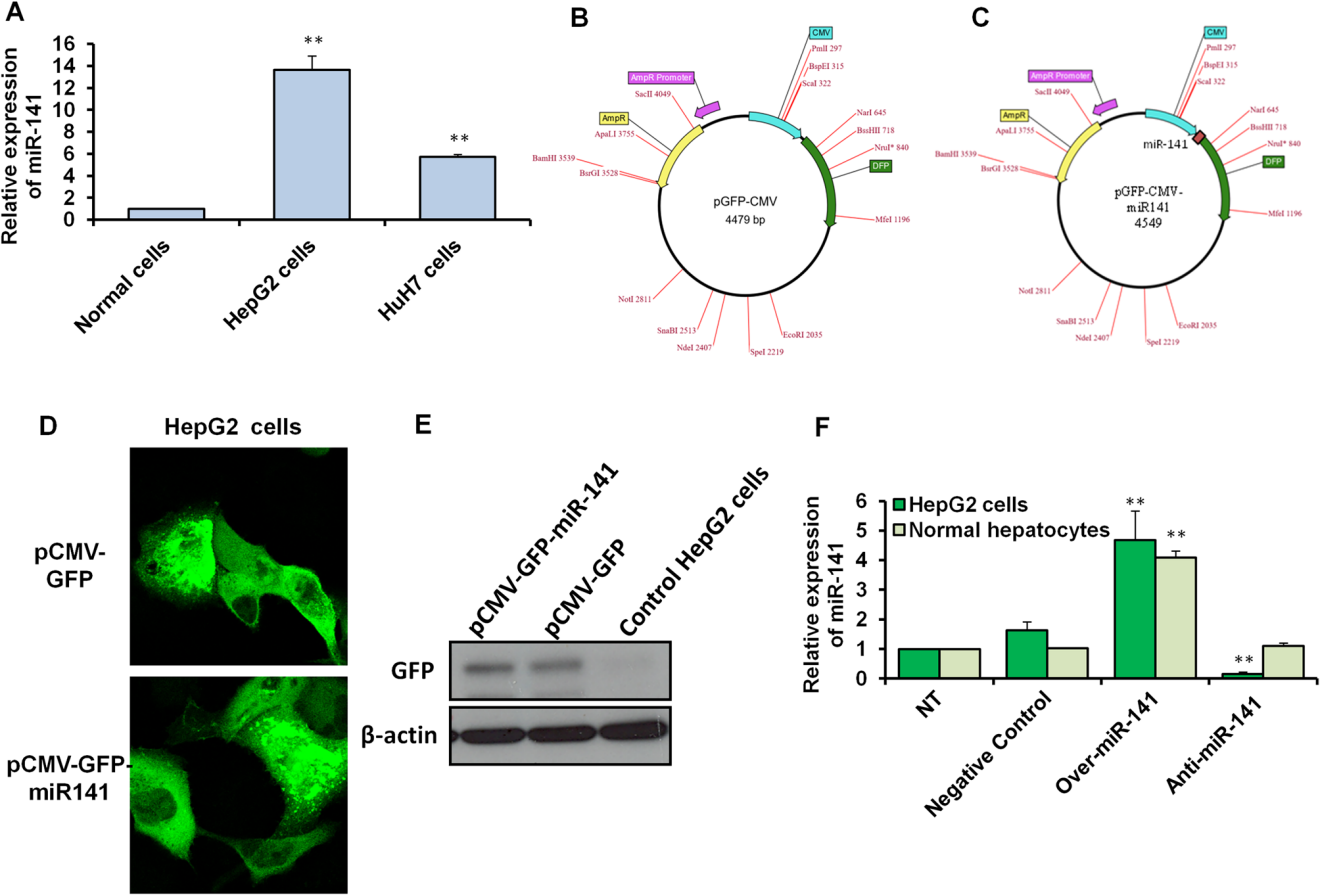
Establishment of miR-141 overexpression system and its levels in liver cancer cell lines and normal hepatocytes. (**A**) Quantification of miR-141expression level indicated by fold changes in liver cancer cell lines, including HepG2 cells and HuH7 cells compared with normal hepatocyte cells using qRT- PCR. (**B** and **C**) Schematic representation of pCMV-miR-141-GFP and empty construct map showed the cloned full-length miR-141 downstream of CMV promoter cassette using online Molbiotools. (**D**) Immunofluorescent assay of transfected HepG2 cells with the miR-141 overexpression vector tagged with GFP that markedly represents the GFP signaling in transfected cells. (**E**) Immunoblotting assay of transected HepG2 cells reveals the protein expression profile of GFP in transfected cells. The protein expression of β-actin severed as loading control. (**F**) The relative expression level of miR-141 in transfected HepG2 cells and normal hepatocytes in comparison with nontransfected cells (NT) and control-transfected cells. Error panels indicate the standard deviation (STD) of three independent experiments. Student two-tailed *t*-test used for statistical analysis, (*) indicates *P-* values ≤ 0.05, (**) indicates *P* ≤ 0.01, and (***) indicates *P* ≤ 0.001.

The expression level of miR-141 was first measured in different liver cancer cells, including HepG2 and HuH7 cells, compared with its expression level in the normal hepatocytes. As shown in Fig. 1A and Table 2, the relative expression of miR-141 significantly increased in both cell lines and reached a 12-fold change in HepG2 cells compared with the normal cells. To determine whether miR-141 plays a role in HepG2 cell proliferation, we constructed a miR-141 overexpression vector using a pCMV-GFP vector with a CMV promoter and original enhanced GFP sequences. The whole sequence of miR-141 was inserted downstream of the CMV promoter region and upstream of GFP original sequences (Fig. 1B and C). The new construct pCMV-GFP-miR-141 was then used to transfect HepG2 cells compared with the others transfected with specific miR-141 inhibitor. The transfected cells with miR-141 overexpression vector showed a potent expression of GFP indicated by immunofluorescent microscope in green signals (Fig. 1D). The successful expression of GFP protein was further confirmed by an immunoblotting assay that showed an evident protein band in cells transfected with either pCMV-GFP vector or pCMV-GFP-miR-141 overexpression vector (Fig. 1E and Supp. Fig. 1). The miR-141 overexpression vector was used to transfect HepG2 cells and normal hepatocytes to validate the overexpression of miR-141, parallel with the transfection with a specific inhibitor antagonist miR-141. The result showed a significant upregulation of miR-141 in cells transfected with the overexpression vector while showing a significant reduction in its expression level in HepG2 cells compared with normal hepatocytes, non-treated and control transfected cells (Fig. 1F). These data confirm the upregulation of miR-141 in HepG2 and HuH7 cell lines in comparison with its expression in the normal hepatocytes and validated the establishment of miR-141 overexpression construct tagged with GFP downstream of CMV promoter.

**Table 2:** Quantification analysis of miR-141 in liver cancer cell lines.

Conditions	Expression fold changes	STD	*P values*
Normal hepatocytes	1	00.00	
HepG2 cells	10.3**	0.72	0.002
HuH7 cells	6.23**	0.85	0.010

STD: standard deviation.

**Indicates high significant *P* values ≤ 0.01.

### Microarray analysis represents miR-141-regulated genes, including the KLK10 and TNFSF-15 gene in HepG2 cells

**Figure 2:**
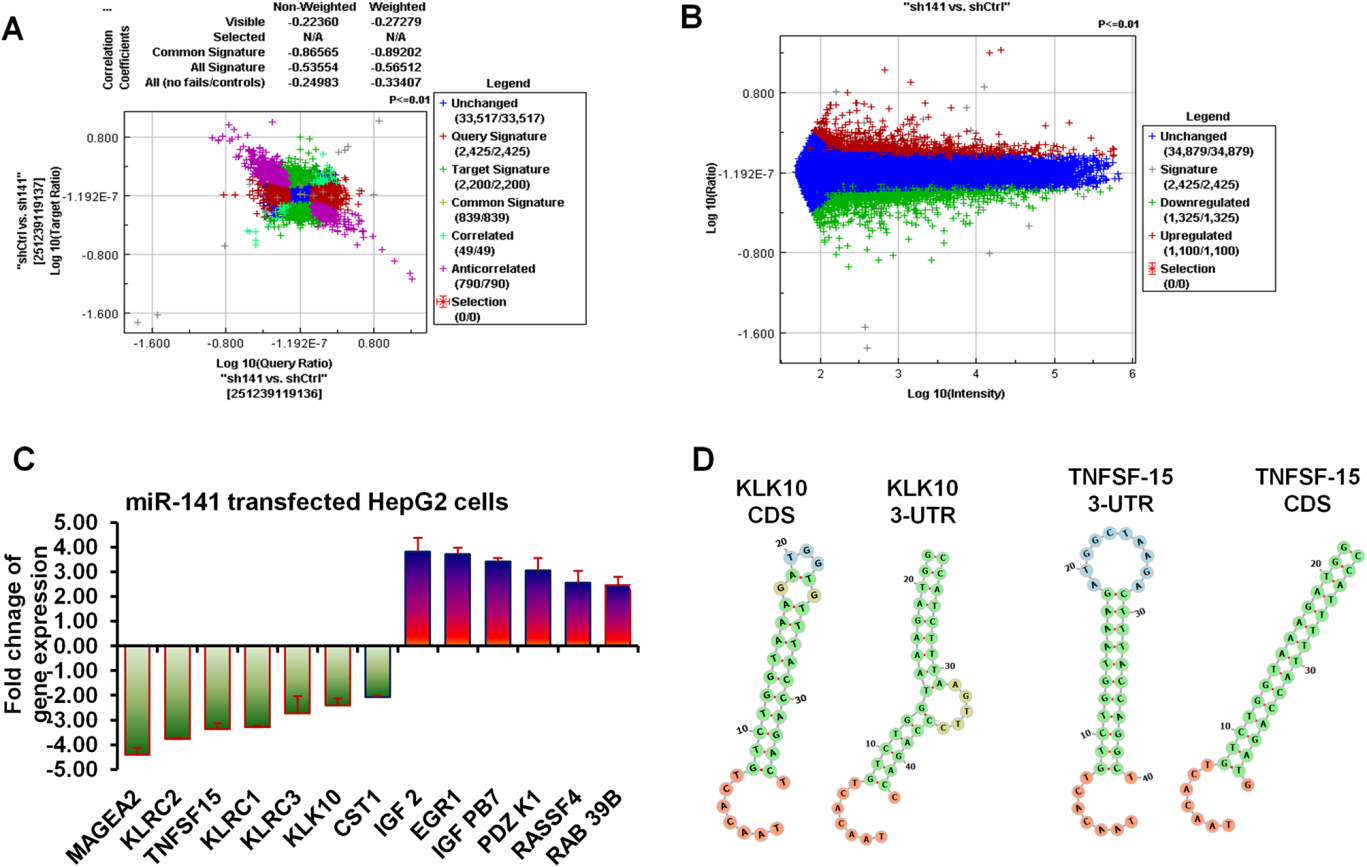
Microarray analysis of transfected HepG2 cells with miR-141 overexpression and its predicted targets. (**A**) Microarray analysis of gene expression in HepG2 cells transfected with miR-141 overexpression vs. cells transfected with the control vector using a miRNA library from Exiqon. (**B**) Microarray analysis of gene expression in HepG2 cells transfected with miR-141 overexpression vs. cells transfected with the control vector using oligonucleotides from Ambion. The blue color indicates the sustained gene expression, the red color indicates the upregulated genes, and the green color indicates the downregulated genes. (**C**) The expression of the most relevant genes to liver cancer indicates the upregulated genes in red columns and downregulated genes in green. Error bars indicate the STD between Exiqon and Ambion data. (**D**) The potential seeding regions of hsa-miR-141 within the coding sequences and 3-UTR of KLK10 and TNFSF-15 gene sequences that carried out *in-silico* by *miRWalk* online tool.

As presented in Fig. 2A and B, the microarray data of transfected HepG2 cells with pCMV-GFP-miR-141 construct showed that dozens of mRNA whose expression was negatively affected by the presence of high levels of miR-141 indicated by Exiqon and Ambion chips. However, thousands of mRNA molecules were not affected by the level of miR-141 in HepG2 cells. In contrast, the expression of many genes was elevated in the presence of these high levels of miR-141. Notably, mRNAs regulated by miR-141 were highly enriched for transcripts subject to cancer repression, and the miR-141-dependent regulation of many curtail tumor suppressor mRNAs, including the MAGEA2, KLRC1, KLRC3, and KLK10 was approved (Fig. 2C). Alternatively, the most increased RNAs were linked with cancer progression and development, such as EGR1, RAS39B, and TNFSF-15 as tumor necrosis (Fig. 2C). This data indicates the possible integration of miR-141 in liver cancer progression and suggests the regulatory role of miR-141 in regulating *KLK10* and *TNFSF-15* gene expression in HepG2 cells. To check whether miR-141 interferes with identified genes by microarray analysis, the *in-silico miRWalk* tool was used to detect the possible binding site of miR-141 and targeted sequences. The seed region of miRNA is a highly conserved sequence with 8 nucleotides in length starting at the 5′ and ending toward the 3′ ends of miRNA. The seed region enables miRNA to be classified into families and species. The *miRWalk* analysis is based on the seed match occurs between miRNA and mRNA at the 3′ end of the miRNA sequence that allowing the strong pairing of the seed region with the targeted genes. To measure the interaction between the miRNA and its target mRNA, minimum free energy is considered. MiRNA: mRNA binding increases when the free energy is low. Negative free energy reactions have less energy available for future reactions, resulting in a stable interaction between miRNA and mRNA. Docking interaction presented in Fig. 2D indicates the binding site of miR-141 within the coding sequences (CDS) and 3-untranslated region (3-UTR) of *KLK10* and *TNFSF-15* targeted sequences. The binding sites, docking score, and required energy are presented in Table 3. The microarray analysis and bioinformatics data indicate the possible regulation of *KLK10* and *TNFSF-15* gene expression by miR-141.

**Table 3:** The prediction information between miR-141 and targeted genes identified by microarray analysis using *in-silico miRWalk* tool.

Ref. no	Gene symbol	Score	N pairings	Position	Binding site	Energy
NM_002776	KLK 10	0.92	15	3-URT	1000–1050	–18
NM_002776	KLK 10	0.90	10	CDS	1650–1700	–18
NM_005118	TNFSF15	0.88	11	3-URT	115–127	–21
NM_005118	TNFSF15	0.85	15	CDS	890–905	–21
NM_001321402	MAGEA2B	0.85	12	5-UTR	235–247	–19
NM_007333	KLRC3	0.84	16	CDC	675–700	–17
NM_014060	MCTS1	1.0	14	3-URT	3668–3684	–17

N: number; 3-UTR: 3-untranslated region; CDS: coding sequences.

### Regulation of HepG2 cell proliferation and viability by miR-141expression level

**Figure 3:**
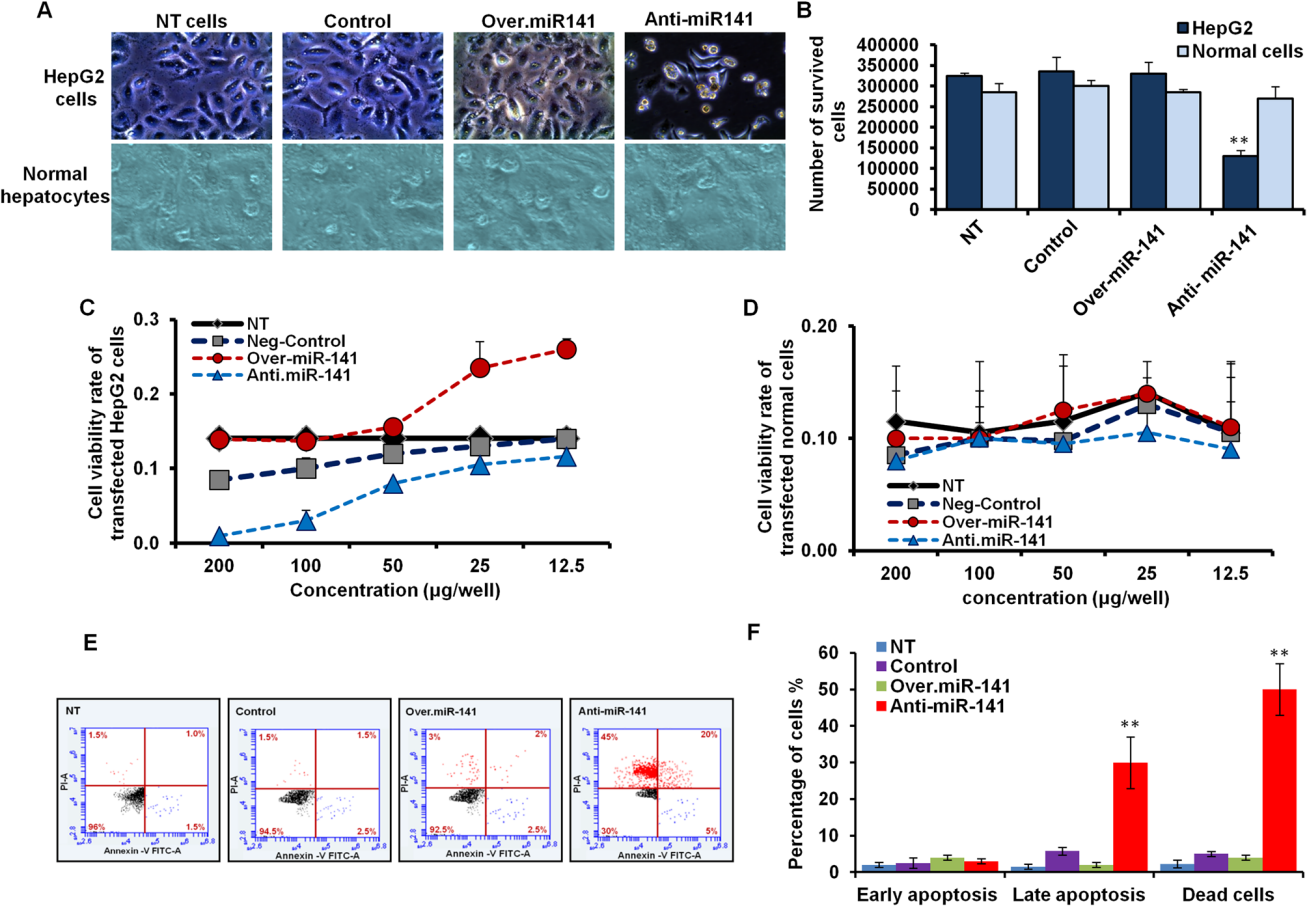
The influence of miR-141 in cell viability and cyotoxicity in transfected HepG2 cells and normal hepatocytes. (**A**) The cell morphology of HepG2 cells and normal hepatocytes indicated by inverted microscope upon 48 h of transfection with either miR-141 overexpression vector or an inhibitor antagonist miR-141 compared with control-transfected and untreated cells (NT). (**B**) After transfection with the miR-141 inhibitor or the overexpression vector, the number of living normal hepatocytes and HepG2 cells. (**C**) Cell viability rate of transfected HepG2 cells with different concentrations of the miR-141 inhibitor or the overexpression vector indicated by the absorbance rate of treated cells with MTT agent. (**D**) Cell viability rate of transfected normal hepatocyte cells with different concentrations of the miR-141 inhibitor or the overexpression vector indicated by the absorbance rate of treated cells with MTT agent (**E**) HepG2 cells were transfected with either miR-141 overexpression vector or inhibitor for 48 h, and then cells were stained with (Annexin V+/Propidium *Iodide* (PI)). The early apoptotic cells and late dead cells were monitored using flow cytometry. (**F**) The percentage of transfected cells with positive signals for early or late apoptosis and the percentage of dead cells indicated by flow cytometric assay. Error panels indicate the standard deviation (STD) of three independent experiments. Student two-tailed *t*-test used for statistical analysis**, **(*) indicates *P-values* ≤ 0.05, (**) indicates *P* ≤ 0.01, and (***) indicates *P* ≤ 0.001.

To investigate the effect of miR-141 expression level on HepG2 cell viability compared with the normal hepatocytes, the miR-141 overexpression vector or specific inhibitor was individually transfected in either HepG2 cell line or normal cells. Unlike the normal hepatocytes, HepG2 cells transfected with miR-141 overexpression vector showed accelerated growth rates and large-scale cell congestion, evidenced by cell morphology presented in Fig. 3A. In contrast, the cell morphology of HepG2 cells transfected with miR-141 specific inhibitor showed marked alteration in the growth of cells and large areas devoid of any development of cancer cells (Fig. 3A). Furthermore, the number of living cells significantly reduced in HepG2 cells transfected with the inhibitor antagonist miR-141 while showing no significant differences compared with control-transfected cells and untreated cells (Fig. 3B and Table 4). Notably, the normal hepatocytes showed negligible differentiation in the number of living cells upon transfection with either miR-141 overexpression vector or a specific inhibitor compared with the control-transfected cells (Fig. 3C and Table 5). Similarly, the cell viability rate of HepG2 cells transfected with an overexpression vector of miR-141 showed neglected differentiation compared with control-transfected cells. In contrast, the cell viability rate of transfected cells with miR-141 specific inhibitor strongly decreased in dose-dependent inhibitor concentrations (Fig. 3C). On the contrary, the cell viability rate of normal hepatocytes showed a constant and stable level in transfected cells as well as the control-transfected cells, indicating the neutral effect of miR-141 level in normal cell growth (Fig. 3D). To check whether the inhibition of miR-141 could modulate programmed cell death (PCD) in transfected HepG2 cells, we monitored the cells with early and late apoptotic signaling in addition to the dead cells by Annexin V assay using the flow cytometric. As presented in Fig. 3E, our findings revealed that less apoptotic signaling and a lower percentage of dead cells were detected in non-treated (NT) HepG2 cells, control transfected cells, and cells transfected with miR-141 overexpression vector. In contrast, the transfection with the inhibitor antagonist miR-141 showed increasing levels of dead cells and activated apoptotic signaling in approximately 50% of stained cells (Fig. 3E and F). These data demonstrate the potential regulatory role of miR-141 expression level in regulating HepG2 cell proliferation and PCD.

**Table 4:** Number of survived HepG2 cells in 48 h post transfection.

	UT	Control	Anti-miR-141	miR-141 overexpression
Mean	294,000	232,000	98,000**	319,000
STD	8485.281	67,882.25	2828.427	12,727.92
*P values*		0.328	0.010	0.147

UT: untreated cells; STD: Standard deviation of three independent experiments.

**Indicates high significant *P* values ≤ 0.01.

**Table 5:** Number of normal cells survived upon 48 h post transfection.

	UT	Control	Anti-miR-141	miR-141 overexpression
Mean	285,000	3,000,000	285,000	270,000
STD	21,213.2	14,142.14	7071.068	28,284.27
*P values*		0.49	1	0.60

UT: untreated cells; STD: standard deviation of three independent experiments.

### Relationship between miR-141 expression level and expression profile of *KLK10* and *TNFSF-15* in HepG2 cells

**Figure 4:**
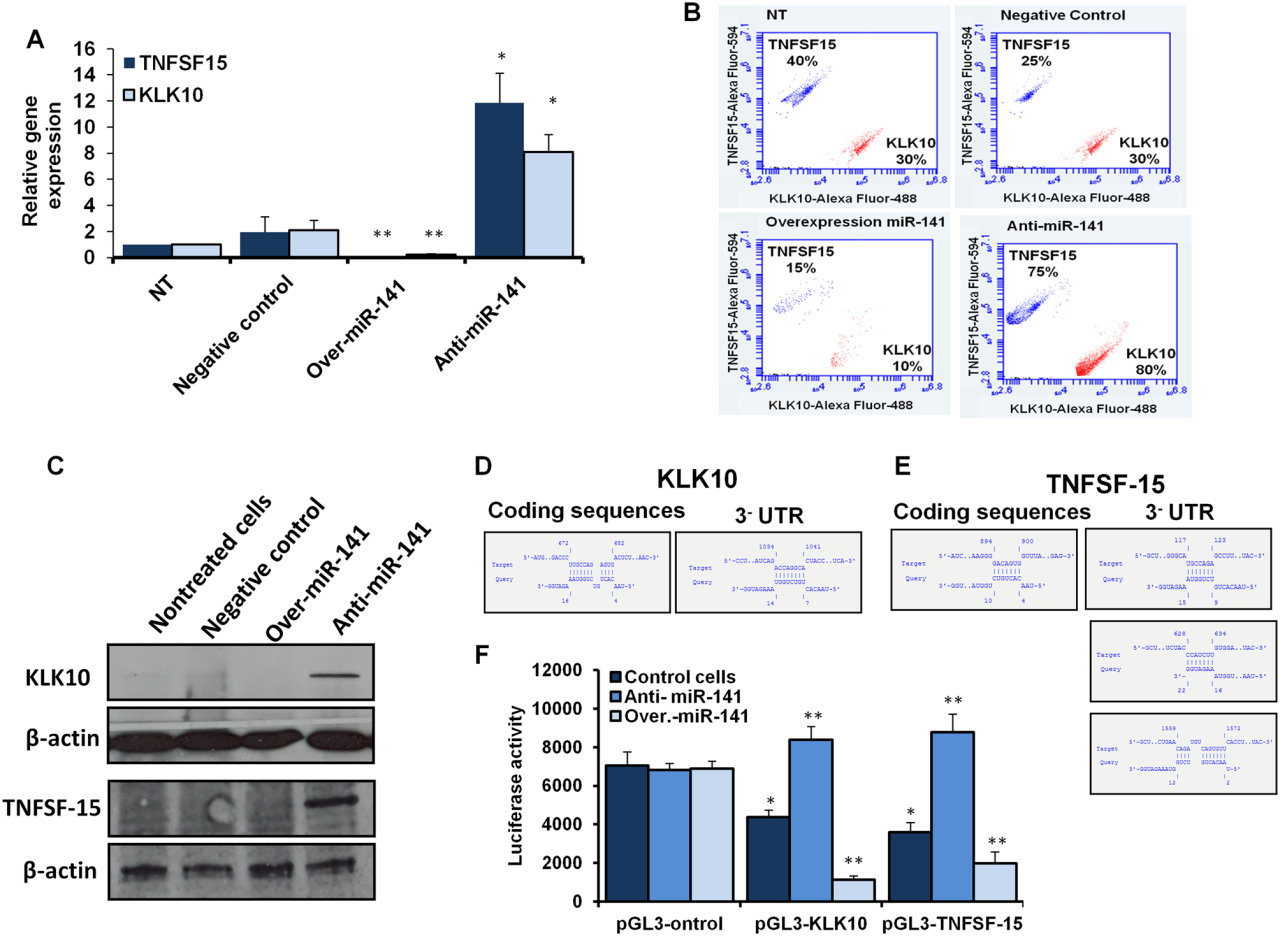
The correlation between the level of miR-141 and KLK10 and TNFSF-15 on gene expression level and protein level in HepG2 cells. (**A**) The relative gene expression of KLK10 and TNFSF-15 in HepG2 cells transfected with the inhibitor against miR-141 and miR-141 overexpression vector indicated by fold change compared with control-transfected and nontreated cells using qRT-PCR. Error bars indicate the STD of three independent experiments. Student two-tailed *t*-test used for statistical analysis**, **(*) indicates *P-values* ≤ 0.05, and (**) indicates *P* ≤ 0.01. (**B**) Flow cytometric assay quantifies the kinetic proteins expression profile of KLK10 (the blue dots) and TNFSF-15 (the red dots) in transfected cells compared with control-transfected and nontreated (NT) cells. (**C**) Western blot analysis shows the protein expression of KLK10 and TNF SF15 in transfected cells compared to control-transfected and nontreated cells. β-actin expression severed as an internal control. (**D** and **E**) Schematic representation of miR-141 binding sites and seeding regions (SR) in KLK10 and TNFSF-15 gene sequence indicated by IntaRNA program. (**F**) In HepG2 cells pre-transfected with miR-141 overexpressing vector or specific inhibitor, the luciferase activities upon cotransfection with luciferase reporter constructs, pGL3-KLK10 or pGL3-TNFSH-15 compared with cells cotransfected with pGL3-control vector. Error bars reveal the STD of three replicates. Student two-tailed *t*-test used for statistical analysis, (*) indicates *P-*values ≤ 0.05, and (**) indicates *P* ≤ 0.01.

To check the possible regulation of *KLK10* and *TNFSF-15* gene expression by miR-141, their expression profile was quantified in transfected HepG2 cells with the antagonist miR-141 and miR-141 overexpression vector using qRT-PCR, flow cytometry, and western blot. Consequently, the relative gene expression of both *KLK10* and *TNFSF-15* was strongly reduced in miR-141 transduced HepG2 cells, while their expression increased in cells transfected with miR-141 inhibitor (Fig. 4A and Table 6). Furthermore, the kinetic protein expression of KLK10 and TNF SF15 markedly depleted in cells transduced miR-141, indicated by flow cytometry, since their expression has been detected in only 10% and 15% of stained cells respectively (Fig. 4B). However, the protein expression profile of both KLK10 and TNF SF-15 showed an evident expression in more than 80% and 75% of stained cells transfected with the inhibitor antagonist miR-141 compared with control-transfected cells, as presented in Fig. 4B. Taken together, these results indicate that miR-141 can regulate the expression profile of both genes, *KLK10* and *TNFSF-15* which play an essential role in the regulation of HCC development. Additionally, the protein expression of KLK-10 and TNFSF-15 was further confirmed via western blot. Western blot analysis showed a potent expression of KLK-10 and TNF SF-15 in cells transfected with the respective inhibitor of miR-141. In contrast, their expression was strongly depleted in cells transfected with miR-141 overexpression vector, control-transfected, and nontreated cells (Fig. 4C and Supp. Figs. 2 and 3). Interestingly, various binding sites have been detected in the coding sequence of *KLK10,* which interfere with mature miR-141 with the required energy − 8.39, − 5.75, and − 0.21, respectively (Fig. 4D and Table 7). TNFSF-15 showed a binding site with miR-141 in its coding sequence with required energy − 5.42 (Fig. 4E and Table 7). Based on this, we cloned the seeding region of potentially targeted genes in the pGL3 luciferase reporter vector with SV40 promoter to validate the direct interaction between miR-141 and *KLK-10* and *TNFSF-15* coding sequences (Supp. Fig. 4). Interestingly, the luciferase activity significantly decreased in HepG2 cells cotransfected with miR-141 overexpression vector and luciferase reporter construct (pGL3-KLK-10 or pGL3-TNFSH-15). Meanwhile, the luciferase activities markedly increased in cells cotransfected with specific inhibitor antagonist miR-141 and pGL3-KLK-10 or pGL3-TNFSH-15 constructs (Fig. 4F). These findings indicate the first evidence concerning the regulation of *KLK10* and *TNFSF-15* gene expression by miR-141 in hepatic cancer cells.

**Table 6:** Quantification analysis of *KLK 10* and *TNFSF-15* in transfected HepG2 cells.

Genes	Condition	Expression fold changes	STD	*P*-values
KLK10	NT	1.00	0.00	
	Control	1.15	0.17	0.35
	Anti-miR-141	6.51*	1.37	0.025
	miR-141 over	0.20**	0.12	0.010
TNFSF-15	NT	1	0.00	
	Control	1.26	0.44	0.49
	Anti-miR-141	4.82**	0.59	0.010
	miR-141 over	0.28**	0.01	0.002

NT: nontreated cells; STD: Standard deviation of three independent experiments.

*Indicates significant *P* values ≤ 0.05.

**Indicates high significant *P* values ≤ 0.01.

**Table 7:** Detailed of selected interaction between miR-141 and targeted genes.

Target (T)	Start (T)	End (T)	Position	Query (Q)	Start (Q)	End (Q)	Energy
KLK10	672	682	CDS	Hsa-miR-141	4	16	− 3.09
KLK10	1034	1041	3-UTR	Hsa-miR-141	7	14	− 5.75
TNFSF-15	894	900	CDS	Hsa-miR-141	4	10	− 3.53
TNFSF-15	117	123	3-UTR	Hsa-miR-141	9	15	− 4.06
TNFSF-15	628	634	3-UTR	Hsa-miR-141	16	22	− 3.59
TNFSF-15	1559	1572	3-UTR	Hsa-miR-141	2	12	− 2.42

CDS: coding sequence; 3-UTR: 3-untranslated region.

### Deception of miR-141successfully modifies the production of TNF-α, IL-4, IL-6, and IL-10 in transfected HepG2 cells

**Figure 5:**
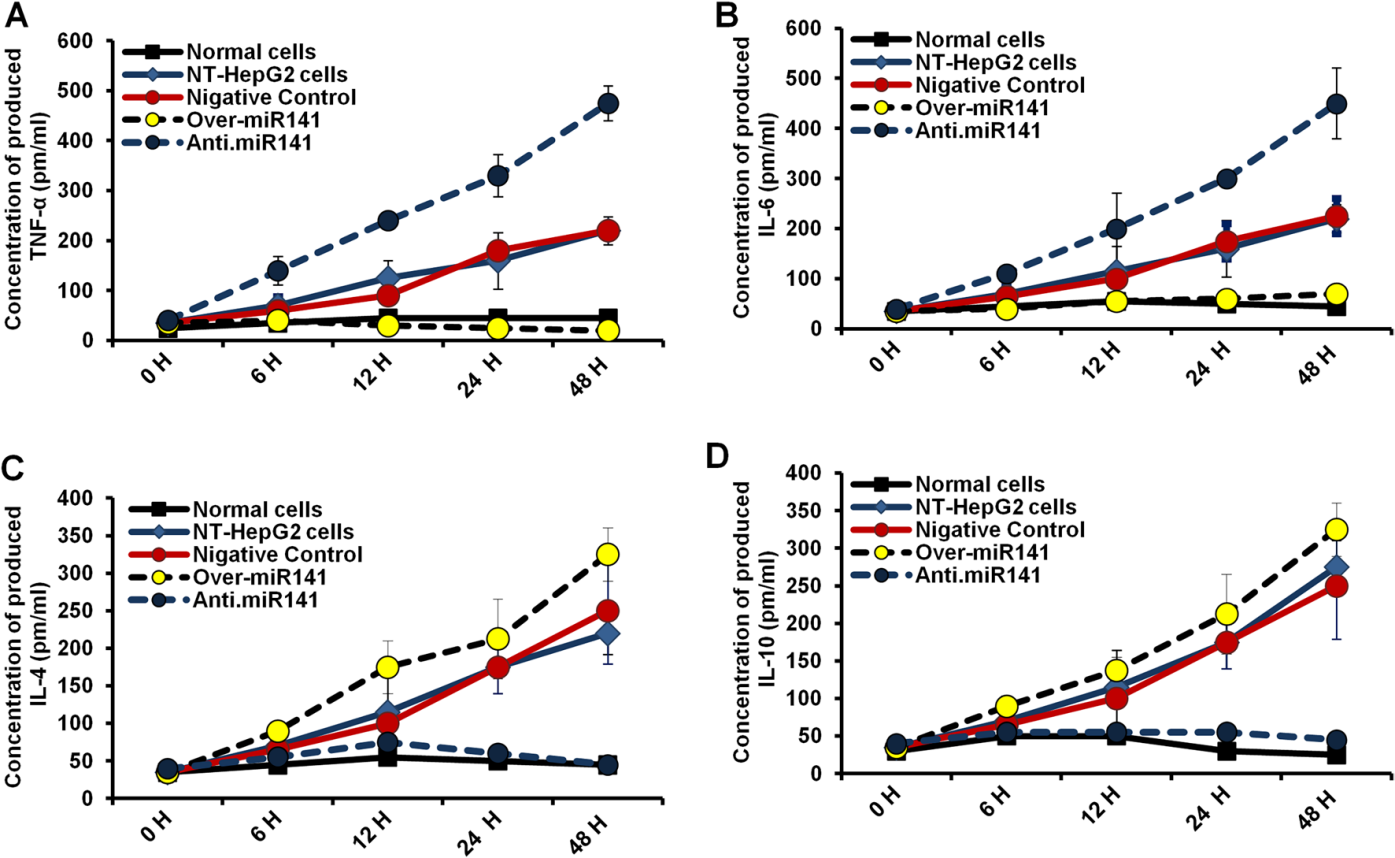
Levels of produced inflammatory cytokines in transfected HepG2 cells. (**A**) The concentration of TGF-β (pm/ml) produced in the fluid media of transfected HepG2 cells in response to the expression level of miR-141 at the indicated time points and compared with nontreated cells (NT) and normal hepatocytes. (**B**) The concentration of IL-8 in the culture media of transfected HepG2 cells simultaneously post-transfection compared with NT cells and normal hepatocytes. (**C** and **D**) At the same time points and compared with NT cells and normal hepatocytes, the concentration of IL-4 and IL-10 in the fluid media of transfected HepG2 cells points to post-transfection, respectively. The Expression rate of IL-4 and IL-10 was elevated remarkably with the overexpression of miR-141, while their expression decreased with transfection with anti-miR141.

To investigate the relationship between miR-141 expression level and the proinflammatory cytokines, including TNF-α and IL-6, the concentration of TNF-α and IL-6 were monitored in transfected HepG2 cells in a time-course experiment. As shown in Fig. 5A, the amount of produced TNF-α dramatically decreased in cells transfected with miR-141 overextension vector in a time-dependent manner. In contrast, its delivered level was markedly increased in cells transfected with miR-141 inhibitor and reached 500 pm/ml 48 h post-transfection. Likewise, the level of produced IL-6 significantly decreased in miR-141 transduced cells while markedly increasing up to 500 pm/ml in cells transfected with the inhibitor antagonist miR-141 (Fig. 5B). Meanwhile, the concentration of both TNF-α and IL-6 showed negligible differentiation in non-transfected and control-transfected cells. On the other hand, the expression of IL-4 and IL-10 as anti-inflammatory cytokines was investigated as well. In a time-course experiment, the concentration of secreted IL-4 and IL-10 was monitored in transfected HepG2 cells. As shown in Fig. 5C and D, the amount of produced IL-4 and IL-10 was markedly decreased in cells transfected with the inhibitor antagonist miR-141 in a time-dependent manner. In the same context, the level of produced IL-4 and IL-10 significantly increased in cells transfected with miR-141 overexpression vector and control transfected cells (Fig. 5C and D). These data indicate that inhibition of miR-141 expression can adjust the production level of both proinflammatory and anti-inflammatory cytokines, supporting the hypothesis suggesting regulation of cell proliferation without detectable inflammatory events.

### DEN-induced liver cancer in rats reproduced the key in-vitro findings

To investigate the potential upregulation of miR-141 in animal models, liver cancer was induced in Wistar Albino rats by intraperitoneal injection with 200 mg/kg DEN dissolved in saline solution and then followed by a recovery period of 2 weeks. Rats were intravenously injected with CCL4 (0.2 ml/kg i.p. in olive oil) twice weekly as a promoter of liver cancer. Figure 7A illustrates the histological changes in various groups. The control group displayed typical liver structure without any injury signs. DEN-treated rats showed mild swelling after 1 week of injection, which developed into severe hepatic damage in 2 weeks. As an indicator of liver cancer, the concentration of TNF-α and IL-10 in rats' serum was increased on day five post-injection and retched gradually at 800 pm/ml on day 20 following injection (Fig. 7B). Likewise, the level of IL-10 increased in DEN-treated rats in a time-dependent manner to a final concentration of 400 pm/ml at day 20 (Fig. 7C). Interestingly, the relative miR-141 expression level increased by four fold-change in 1-week DEN-treated rats and increased by eight fold-change in 2-week DEN-treated rats compared with the control rats (Fig. 7D). Furthermore, the relative gene expression of KLK10 and TNFSF-15 was significantly reduced in rats with liver cancer in a time-dependent manner of DEN treatment (Fig. 7E). These in-vivo findings further confirmed the connection between miR-141 and liver cancer development parallel with the disturbance of KLK10 and TNFSF-15 gene expression.

### The crosslink and network interaction of KLK10 and TNFSF-15 protein

**Figure 6:**
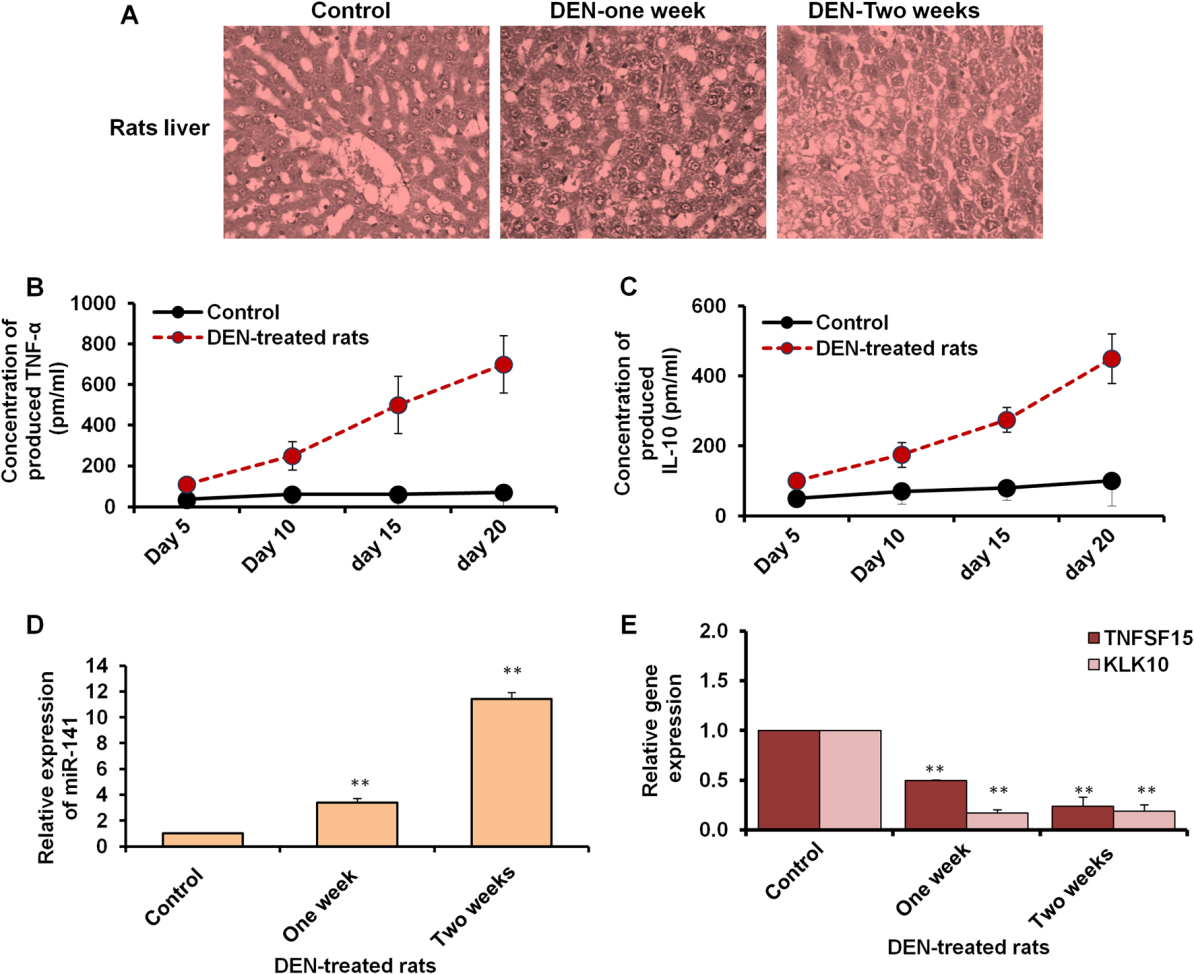
In-vivo study of DEN-induced liver cancer in rats. (**A**) Histological analysis of a liver section of albino rats that were injected with a single dose of DEN either for 1 week, represented a mild swelling or 2 weeks, which revealed hepatic cancer cells in comparison with the liver section of control rats. (**B** and **C**) The level of serum TNF-α and IL-10 in rats with liver cancer in response to DEN treatment at the indicated schedules compared with their concentration in the control rats. Error bars indicate the STD of two sacrificed rats. (**D**) The relative expression level of miR-141 in liver cells of DEN-treated rats for 1 or 2 weeks in comparison with control-treated rats. (**E**) The relative expression of KLK10 and TNFSF-15 genes in liver cells of DEN-treated rats at 1 or 2 weeks compared with control-treated rats. Error bars indicate the STD of two sacrificed rats. Student two-tailed t-test was used to evaluate the significance of indicated values. (**) indicates that the *P* value is ≤ 0.01.

**Figure 7:**
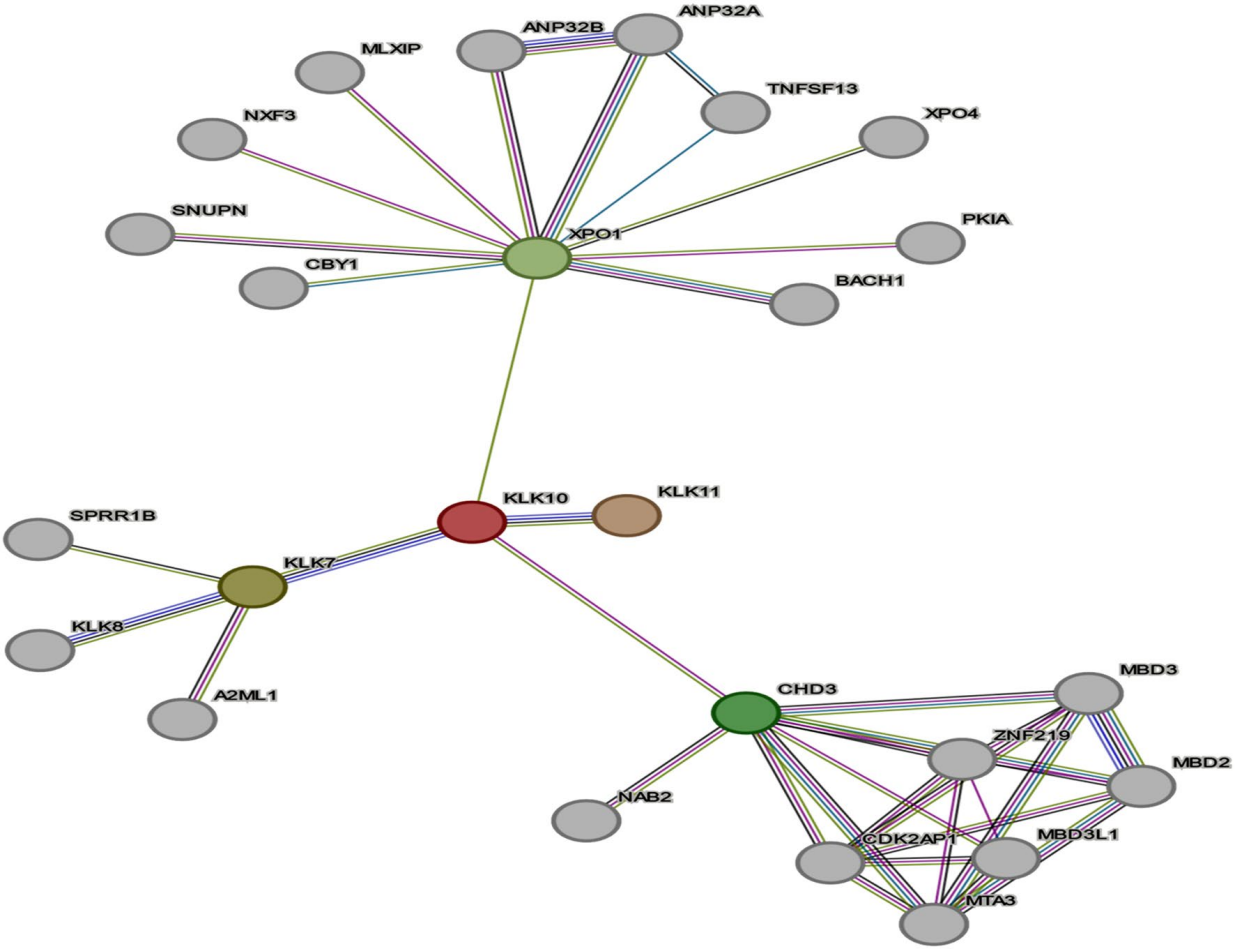
The schematic representation of KLK10 and other proteins interaction. The colored nodes meant the direct interaction/s. The shortlisted targeted genes by miR-141 were selected from the microarray and subjected to Search Tool for the Retrieval of Interacting Genes (STRING) 12 version database analyses. STRING constructed a network model that allows displaying the interaction of all proteins. This analysis is connected to the KEGG and the Gene Ontology (GO) databases to cluster the shortlisted genes as input into various pathways and biological processes. (The red line indicates the presence of fusion evidence, the green line refers to the neighborhood evidence, the blue line shows the co-occurrence evidence, the purple line indicates the experimental evidence, the yellow line refers to the mining evidence, the light blue line suggests the database evidence and black line indicates the co-expression evidence).

The network interaction of KLK10 and TNFSF-15 was studied using STRING 12 version software as presented in Fig. 6. The results showed the protein–protein interactions between the miR-141 targeted proteins. Those are represented in KLK10 and TNFSF-15 which were detected in the microarray analysis in the HepG2 cell line. The analysis was matched in both KEGG and GO databases (Fig. 7). The results showed the interactions between KLK10 and expected proteins such as the KLK protein family, XPO1, and TNFSF-13 factor using the STRING database. In addition, the TNFSF-15 showed wide interactions varied among the members of the TNFSF family (Supp. Fig. 5). Further network interactions of TNFSF-15 with different interleukins and cytokines have been predicted, including TNF, interferon-gamma (IFN-γ), IL-10, FoxP3, and IL-4 (Supp. Fig. 6). Interestingly, the molecular function of most regulated genes by TNFSF-15 is implicated in apoptosis, necrosis, cell proliferation, and viral infection (Supp. Table 1). The predicted network interaction suggests the implication of targeted genes by miR-141 in cell proliferation and PCD.

## Discussion

The significant finding of the present study is that miR-141 inhibits the expression of the *KLK 10* and *TNFSF-15* target genes, promoting liver carcinogenesis. The effect of miR-141 on HepG2 cell proliferation has been studied^36,37^. However, little is known concerning the correlation between the targeted genes by miR-141 in liver cancer, which is needed for extensive studies. In this study, we aimed to identify new targeted genes by miR-141. As a member of the miR-200 family, which consists of miR-200a, 200b, miR-200c, and miR-429^38^, miR-141 is typically expressed in various malignant tumors, contributing to many cellular processes such as cell proliferation, epithelial-mesenchymal transition (EMT), invasion, metastasis, and drug resistance. Here we further confirmed the upregulation of miR-141 in liver cancer cell lines compared with normal hepatocytes. In the case of miR-141 inhibition by an anti-miR-141, HepG2 cell viability showed a significant decrease as it reached 14,000 living cells with 70% less compared to the non-treated cells. Afterward, the overexpression of miR-141 showed the increase of the living cells to the limit of the negative control and close to the non-treated cells. Owing to the morphology of the HepG2 cells, it showed no difference between the non-treated cells and the negative control cells, while it deteriorated in the case of transfection by anti-miR-141. Such deterioration lessened in the case of transfection with an overexpression vector of miR-141, which showed detectable differences in both shape and number of the cells. In the same context, the transfection with anti-miR141 was performed in HepG2 cells to detect its effect on cell viability rate whenever treated with different concentrations of miR-141 inhibitors. As potentially targeted by miR-141, KLK-10 expression profile showed markedly depletion in cells transfected with miR-141 overexpression vector, while showing increasing levels in cells transfected with the inhibitor antagonist miR-141. Additionally, the role of miR-141 in regulating the expression of TNFSF-15 was determined.TNFSF-15 is an exceptional cytokine that plays a role in the modulation of vascular homeostasis and inflammation. TNFSF15 is overexpressed in established vasculature and down-regulated in neovascularization, including cancers and wounds. Evidence indicated that TNFSF15 disturbs endothelial cell differentiation^16^. Importantly, TNF is a pro-inflammatory cytokine that functions as a double-dealer in carcinogenesis. Therefore, it may be considered a cancer promoter by activating nuclear factor κB (NF-κB), an anti-apoptotic signaling pathway. However, TNF may contribute to cell death via c-Jun N-terminal kinase (JNK) signaling pathway^39^. Additionally, TNF plays a pivotal role in liver carcinogenesis as it activates hepatocyte apoptosis and necrosis as a biological response; in contrast, it may lead to liver inflammation as well after its generation. Importantly, the anti-TNF therapies are strongly proposed in liver cancer treatment at its specific receptors^15^. TNFSF-15 is considered a pro-inflammatory cytokine as its implication in autoimmune diseases. However, it is involved in both hepatocyte necrosis and apoptosis, besides liver inflammation and HCC progression^40^. Deficiency of the TNFR-dependent anti-apoptotic NFκB signaling pathway is essential for the induction of compensatory proliferation of live hepatocytes, which results in the development of HCC^15^. In the same context, in colon cancer, miR-141 and miR-425-3p activate mutations in KRAS in Colorectal cancer (CRC) patients. Additionally, the miR-200 family binds to RASSF2 in the MAPK/ERK signaling pathway which support their oncogenic role^8,10,15^. Moreover, miR-141 can target the STAT4 gene expression, leading to liver cancer cell progression^41^. The elevation of serum TNF concentration was reported in various cancer patients when its expression was higher in various pre-neoplastic and tumor tissues^42^. TNF expression is elevated in various types of cancer like cervical cancer, prostate cancer, and breast cancer^43^. Alternatively, TNF concentration may be decreased during chemotherapy either in breast or prostate cancer patients, which correlates with serum TNF level as a chemotherapy response and confirms its role in cancer development. Further, TNFSF-15 expression is also modulated by IL-1β and chondroitin sulfate in osteoarthritis patients^44^.

Kallikreins are considered a subgroup of serine proteases with multiple physiological functions. It is well reported that many kallikreins are implicated in carcinogenesis, while others are considered novel cancer biomarkers. KLK10, which is located in a cluster on chromosome 19 and its encoded protein, suppresses tumorigenesis in both breast and prostate cancers, which results in various transcripts during its splicing, which encodes the same protein as well. The miR-141-3p upregulation could inhibit oral squamous cell carcinoma cell invasion, proliferation, and migration by targeting PBX1 via the JAK2/STAT3 pathway^45^. Interestingly, KLK10 gene has a tumor-suppressive function in distinct types of human cancer^43^. KLK10 downregulated various cancer types like Prostate and breast cancers In addition, in both liver and breast cancer, the drug sensitivity was enhanced by ectopic KLK 10 via reduction of anchorage independent growth^46^. In this study, KLK10 gene expression decreased in HepG2 cells with miR-141 over expressed. However, its expression increased significantly with transfected cells with anti-miR141 which supports the role of Klk10 as a tumor suppressor in HCC. However, it may act as an oncogene as its expression is upregulated in gastrointestinal tumors^47^, however, the mechanism is still not unclear. KLK10, which is highly expressed in human esophageal cancer (EC) is considered as a potential biomarker for EC diagnosis at an early stage. Future studies should examine more tissues and in vivo assays to gain insight into the detailed mechanisms of KLK10-derived EC diagnosis and therapy^11^. KLK10 is expressed in non-small-cell lung cancer (NSCLC) as well^13^. We observed the KLK10 on the mRNA level and the protein level in HepG2 cell and the results were similar in accordance with the level of miRNA-141 expression levels. This may confirm the regulation of KLK10 via miR-141at the transcriptional and post transcriptional level in HCC. In the same context, KLK10 was dysregulated by three miRNAs let-7f, miR-224, or mR-516a, in ovarian cancer^48^ which confirmed by our results as the KLK10 was highly restored in cells transfected with anti-miR-141 which confirm the correlation between klk10 expression on the protein level and miRNA-141 in HCC and its effect on it as a tumor suppressor. In our study, we have checked the possibility of intersection between the KLK10 and TNF15 on the protein level to detect the possible or predicted protein–protein interactions. In addition, we also test the possible pathways that may be involved in it which may direct us to specific biological pathway/s especially in cancer development and initiation using KEGG and KO databases. KEGG is an encyclopedia of genomes and genes that assign functional importance to genes and genomes at the molecular and higher levels. Molecular-level functions are stocked up in the KO (KEGG Orthology) database, where each KO is referees to the functional ortholog of genes and proteins. KEGG is an integrated database resource, including fifteen manually established databases and a computationally generated database in different categories. The databases in the systems information category are PATHWAY, BRITE, and MODULE, which constitute the reference knowledge base for understanding higher-level systemic functions of the cell and the organism, including metabolism, other cellular processes, organism functions, and human diseases. For KLK10, it showed cooccurrence with other KLKs members family like KLK 11, KLK7 and KLK 8. Consequently, the KLK10 was proven to play a crucial role in apoptosis improvement is prostate cancer cells PC3^11^ besides its proliferation suppression. In the same context, the KLK10 showed experimental evidence of interactions with chromodomain helicase DNA binding protein 3 (CHD3)^49^, NGFI-A-binding protein 2 (NAB2), zinc finger protein 219 (ZNF 219), cyclin dependent kinase 2 associated protein 1(CDK2AP1), Methyl-CpG-binding domain proteins 2 (MBD3L1), Methylated DNA binding domain protein 2(MBD2), Methylated DNA binding domain protein 3 (MBD3) and Metastasis-associated protein (MTA3) proteins. On the hand, KLK10 showed neighbored evidence to the XPO1 protein which acts as oncogene and overexpressed in different human cancers^50^. This cooccurrence with KLK1i may reflect the possibility for its involvement in tumorigeneses. Moreover, for the TNFSF15 protein, the STRING database was used to detect the protein interactions. As the STRING databases showed correlation between TNFSF15 with many tumor necrosis factors. For instance, TNFSF-15 predicted to be interaction with TNF receptor superfamily members as TNF receptor superfamily member 21(TNFRSF21), TNFRSF14, TNFRSF8, TNFRSF18, TNFRSF21, TNFRSF6B, TNFRSF1A. Notably, with the analysis of 0.9 confidence interval, the results showed clear correlation evidence between TNFSF15 and TNFRSF25, TNFRSF1A. In addition, the prediction clearly showed correlation between TNFSF15 and many interleukins like, IL-10, IL-5, IL-4, IL-13, IL-18, IL-17A, IL-2RA. It also showed interaction with T-cell surface glycoprotein CD4 (CD4 protein) which is inducted in a crucial role in emerging and enduring effective anti-tumor immunity^51^. Overall, our study provides evidence for the possible interaction of miR-141 as an oncogenic factor in liver cancer cells through posttranscriptionally regulation of KLK10 and TNFSF-15 gene expression and controlling their related signaling pathways. This conclusion may lead to the benefit of the inhibitor antagonist miR-141 as a potential vaccine to protect against liver cancer initiation in addition to the possible exploitation in liver cancer treatment. However, this hypothesis required more investigation of such inhibitor animal models before subjecting them to clinical trials.

## Conclusion

The present study shows the upregulation of miR-141 in liver cancer cell lines, HepG2 and Huh7 cells compared to the normal hepatic cells. Likewise, the increasing level of miR-141 was detected in rats with liver cancer. Microarray analysis of transfected HepG2 cells with miR-141 overexpression vector reveals that miR-141 is implicated in HepG2 cell proliferation and metastasis via targeting KLK10 and TNFSF-15 gene expression profiles. Moreover, miR-141 transduced HepG2 cells further confirms the targeting of KLK10 and TNFSF-15 expression profile at both RNA and protein levels accompanied by decreasing level of produced transforming tumor necrosis factor (TNF-α) and reduced levels of IL-4 and IL-10 production. In contrast, transfected HepG2 cell line with an inhibitor antagonist miR-141 expression provides marked restoration of KLK10 and TNFSF-15 gene expression and increases the production of IL-6. Investigation of the seeding region in the *KLK10* and *TNFSF-15* gene sequences suggested a binding site with miR-141 in their coding sequences. Finally, DEN-induced liver cancer in rats successfully reproduced our key *in-vitro* findings, including the deceasing expression of *KLK10* and *TNFSF-15* genes. These data indicate that miR-141 is involved in liver cancer progression and metastasis via targeting related factors such as KLK10 and TNFSF-15 gene expression.

### Supplementary Information

Supplementary Information.

## Data Availability

The data supporting these findings are included in the main manuscript and supplementary file. The whole microarray results are available from the corresponding author upon reasonable request.

## References

[1] Peng Y, Croce CM (2016). The role of microRNAs in human cancer. Signal Transduct. Target. Ther..

[2] Ghafouri-Fard S (2022). Exploring the role of non-coding RNAs in autophagy. Autophagy.

[3] Shi T (2021). Evaluating the effect of lenvatinib on sorafenib-resistant hepatocellular carcinoma cells. Int. J. Mol. Sci..

[4] Ghafouri-Fard S (2022). A review on the role of PRNCR1 in human disorders with an especial focus on cancer. Pathol. Res. Pract..

[5] Maher E (2020). Hsa-miR-21-mediated cell death and tumor metastases: A potential dual response during colorectal cancer development. Middle East J. Cancer.

[6] Morishita A (2021). Micrornas in the pathogenesis of hepatocellular carcinoma: A review. Cancers.

[7] Gao Y (2016). The roles of MicroRNA-141 in human cancers: From diagnosis to treatment. Cell. Physiol. Biochem..

[8] Huang GL (2019). MiR-200 family and cancer: From a meta-analysis view. Mol. Aspects Med..

[9] Carter JV (2019). The microRNA-200 family acts as an oncogene in colorectal cancer by inhibiting the tumor suppressor RASSF2. Oncol. Lett..

[10] Liu Y (2022). A pair of prognostic biomarkers in triple-negative breast cancer: KLK10 and KLK11 mRNA expression. Life.

[11] Hu J (2015). NES1/KLK10 gene represses proliferation, enhances apoptosis and down-regulates glucose metabolism of PC3 prostate cancer cells. Sci. Rep..

[12] Geng X (2018). Elevated tumor tissue protein expression levels of kallikrein-related peptidases KLK10 and KLK11 are associated with a better prognosis in advanced high-grade serous ovarian cancer patients. Am. J. Cancer Res..

[13] Paliouras M, Borgono C, Diamandis EP (2007). Human tissue kallikreins: The cancer biomarker family. Cancer Lett..

[14] van Loo G, Bertrand MJM (2022). Death by TNF: A road to inflammation. Nat. Rev. Immunol..

[15] Tiegs G, Horst AK (2022). TNF in the liver: Targeting a central player in inflammation. Semin. Immunopathol..

[16] Zhang Z, Li LY (2012). TNFSF15 modulates neovascularization and inflammation. Cancer Microenviron..

[17] Ren X, Fan Y, Shi D, Liu Y (2023). Expression and significance of IL-6 and IL-8 in canine mammary gland tumors. Sci. Rep..

[18] Hirano T (2021). IL-6 in inflammation, autoimmunity and cancer. Int. Immunol..

[19] Kumari N, Dwarakanath BS, Das A, Bhatt AN (2016). Role of interleukin-6 in cancer progression and therapeutic resistance. Tumor Biol..

[20] El-Fadl HMA (2021). Effective targeting of Raf-1 and its associated autophagy by novel extracted peptide for treating breast cancer cells. Front. Oncol..

[21] Khalil H (2024). Amelioration effect of 18β-glycyrrhetinic acid on methylation inhibitors in hepatocarcinogenesis-induced by diethylnitrosamine. Front. Immunol..

[22] Abd El Maksoud AI (2020). Methylomic changes of autophagy-related genes by legionella effector Lpg2936 in infected macrophages. Front. Cell Dev. Biol..

[23] Guirgis SA, El-Halfawy KA, Alalem M, Khalil H (2023). Legionellapneumophila induces methylomic changes in ten-eleven translocation to ensure bacterial reproduction in human lung epithelial cells. J. Med. Microbiol..

[24] Mohamed E-SA, Bassiouny K, Alshambky AA, Khalil H (2022). Anticancer properties of N, N-dibenzylasparagine as an asparagine (Asp) analog, using colon cancer Caco-2 cell line. Asian Pac. J. Cancer Prev..

[25] Iglesias-Ussel M, Marchionni L, Romerio F (2013). Isolation of microarray-quality RNA from primary human cells after intracellular immunostaining and fluorescence-activated cell sorting. J. Immunol. Methods.

[26] Khalil H, Arfa M, El-Masrey S, El-Sherbini S, Abd-Elaziz A (2017). Single nucleotide polymorphisms of interleukins associated with hepatitis C virus infection in Egypt. J. Infect. Dev. Ctries..

[27] Guirgis SA, El-Halfawy KA, Alalem M, Khalil H (2023). Legionellapneumophila induces methylomic changes in ten-eleven translocation to ensure bacterial reproduction in human lung epithelial cells. J. Med. Microbiol..

[28] Salah A, Sleem R, Abd-Elaziz A, Khalil H (2023). Regulation of NF-κB expression by thymoquinone; a role in regulating pro-inflammatory cytokines and programmed cell death in hepatic cancer cells. Asian Pac. J. Cancer Prev..

[29] Alalem M (2023). Influenza a virus regulates interferon signaling and its associated genes; MxA and STAT3 by cellular miR-141 to ensure viral replication. Virol. J..

[30] Fekry T (2022). Anticancer properties of selenium-enriched oyster culinary-medicinal mushroom, *Pleurotus ostreatus* (Agaricomycetes), in colon cancer in vitro. Int. J. Med. Mushrooms.

[31] Elimam H, El-Say KM, Cybulsky AV, Khalil H (2020). Regulation of autophagy progress via lysosomal depletion by fluvastatin nanoparticle treatment in breast cancer cells. ACS Omega.

[32] Khalil H (2019). Interruption of autophagosome formation in cardiovascular disease, an evidence for protective response of autophagy. Immunol. Investig..

[33] Szklarczyk D (2017). The STRING database in 2017: Quality-controlled protein–protein association networks, made broadly accessible. Nucleic Acids Res..

[34] Rao X, Huang X, Zhou Z, Lin X (2013). An improvement of the2–delta delta CT) method for quantitative real-time polymerase chain reaction data analysis. Biostat. Bioinform. Biomath..

[35] Elawdan KA (2022). Association of vitamin B12/ferritin deficiency in cancer patients with methylomic changes at promotors of TET methylcytosine dioxygenases. Biomark. Med..

[36] Liu Y (2014). MiR-141 suppresses the migration and invasion of HCC cells by targeting Tiam1. PLoS ONE.

[37] Xue J (2014). miR-141 suppresses the growth and metastasis of HCC cells by targeting E2F3. Tumor Biol..

[38] Mao Y (2020). Mechanisms and functions of miR-200 family in hepatocellular carcinoma. Onco. Targets. Ther..

[39] Mitchell CA, Ramessar K, O’Keefe BR (2017). Antiviral lectins: Selective inhibitors of viral entry. Antiviral Res..

[40] Grivennikov SI, Greten FR, Karin M (2010). Immunity, inflammation, and cancer. Cell.

[41] Ma L, Shao H, Chen H, Deng Q (2021). The mechanism of miR-141 regulating the proliferation and metastasis of liver cancer cells by targeting STAT4. J. Oncol..

[42] Mercogliano MF, Bruni S, Elizalde PV, Schillaci R (2020). Tumor necrosis factor α blockade: An opportunity to tackle breast cancer. Front. Oncol..

[43] Sidiropoulos M, Pampalakis G, Sotiropoulou G, Katsaros D, Diamandis EP (2005). Downregulation of human kallikrein 10 (KLK10/NES1) by CpG island hypermethylation in breast, ovarian and prostate cancers. Tumor Biol..

[44] Hui-qin Yang LC (2008). Effects of sinomenine on synovial fluid and serum content of interleukin-1β in rabbits with osteoarthritis. J. Integr. Med..

[45] Li Y (2010). Carcinoembryonic antigen interacts with TGF-β receptor and inhibits TGF-β signaling in colorectal cancers. Cancer Res..

[46] Li L (2015). Upregulated KLK10 inhibits esophageal cancer proliferation and enhances cisplatin sensitivity in vitro. Oncol. Rep..

[47] Tailor PD (2018). Diagnostic and prognostic biomarker potential of kallikrein family genes in different cancer types. Oncotarget.

[48] White NMA (2010). Three dysregulated miRNAs control kallikrein 10 expression and cell proliferation in ovarian cancer. Br. J. Cancer.

[49] Snijders Blok L (2018). CHD3 helicase domain mutations cause a neurodevelopmental syndrome with macrocephaly and impaired speech and language. Nat. Commun..

[50] Azizian NG, Azizian NG, Li Y, Li Y (2020). XPO1-dependent nuclear export as a target for cancer therapy. J. Hematol. Oncol..

[51] Tay RE, Richardson EK, Toh HC (2021). Revisiting the role of CD4+ T cells in cancer immunotherapy—new insights into old paradigms. Cancer Gene Ther..

