# Supplementary material for: MicroRNA-141-regulated KLK10 and TNFSF-15 gene expression in hepatoblastoma cells as a novel mechanism in liver carcinogenesis
Source: Sci Rep. 2024 Jun 12;14:13492. doi: 10.1038/s41598-024-63223-4 (PMC11169620; doi:10.1038/s41598-024-63223-4)
Supplement: Supplementary file 1 — Supplementary Information. [file 41598_2024_63223_MOESM1_ESM.docx]

**Supplementary data**


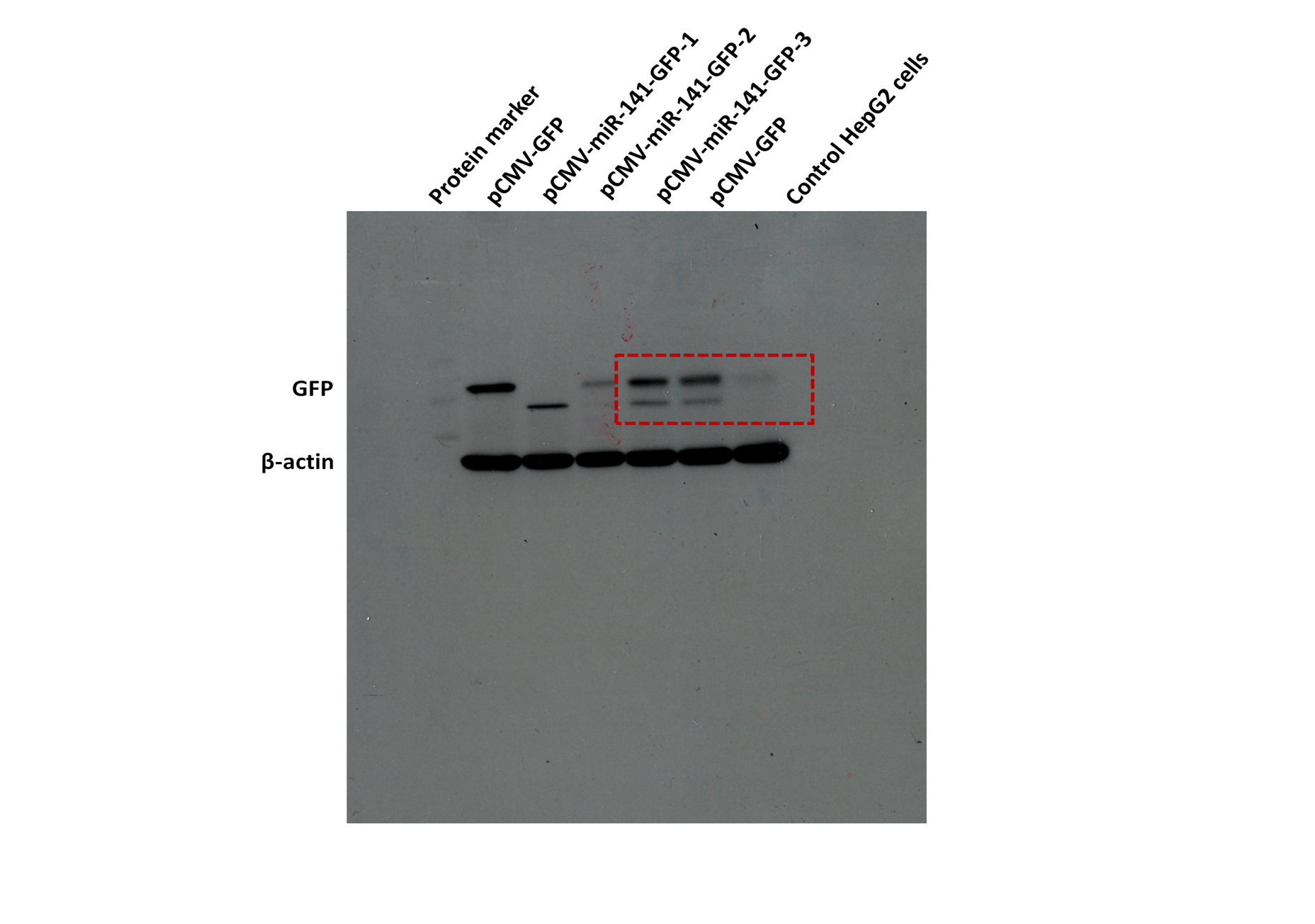


**Supp. Figure 1:** Original uncropped immunoblotting membrane reveal GFP protein expression and β-actin in HepG2 cells that transfected with pCMV-GFP vector or different constructed pCMV-miR-141-GFP vector compared to non-transfected cells using specific antibody antagonist GFP protein. β-actin was introduced as an internal control in all western blot experiments.


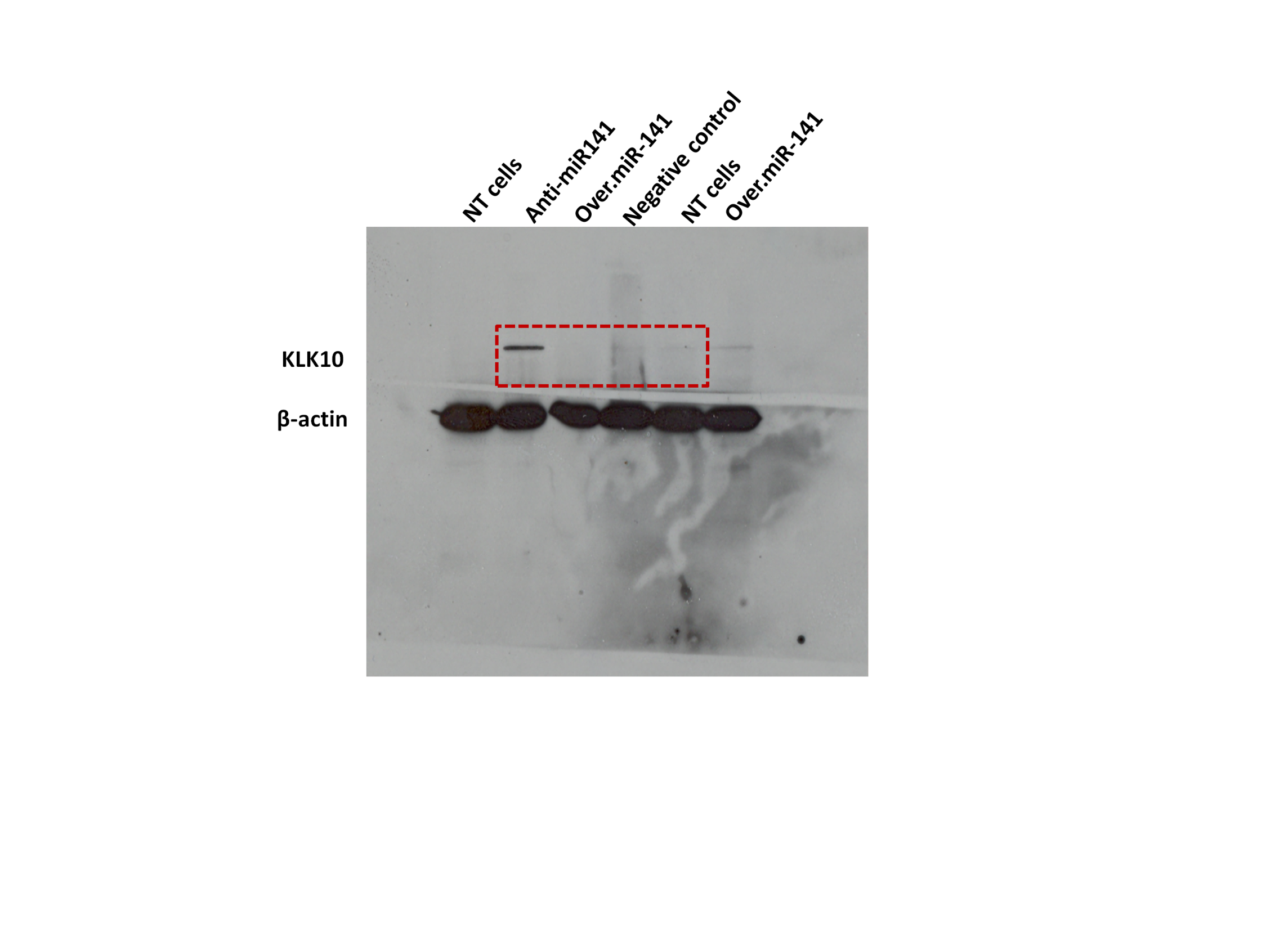


**Supp. Figure 2:** Original uncropped blots of KLK10 immunoblotting membrane reveals the protein levels of KLK10 and β-actin in HepG2 cells that transfected with an inhibitor antagonist miR-141 or the miR-141 overexpression vector compared to non-transfected cells and negative control transfected cells that treated with the same concentration of transfection reagent. β-actin was introduced as an internal control.


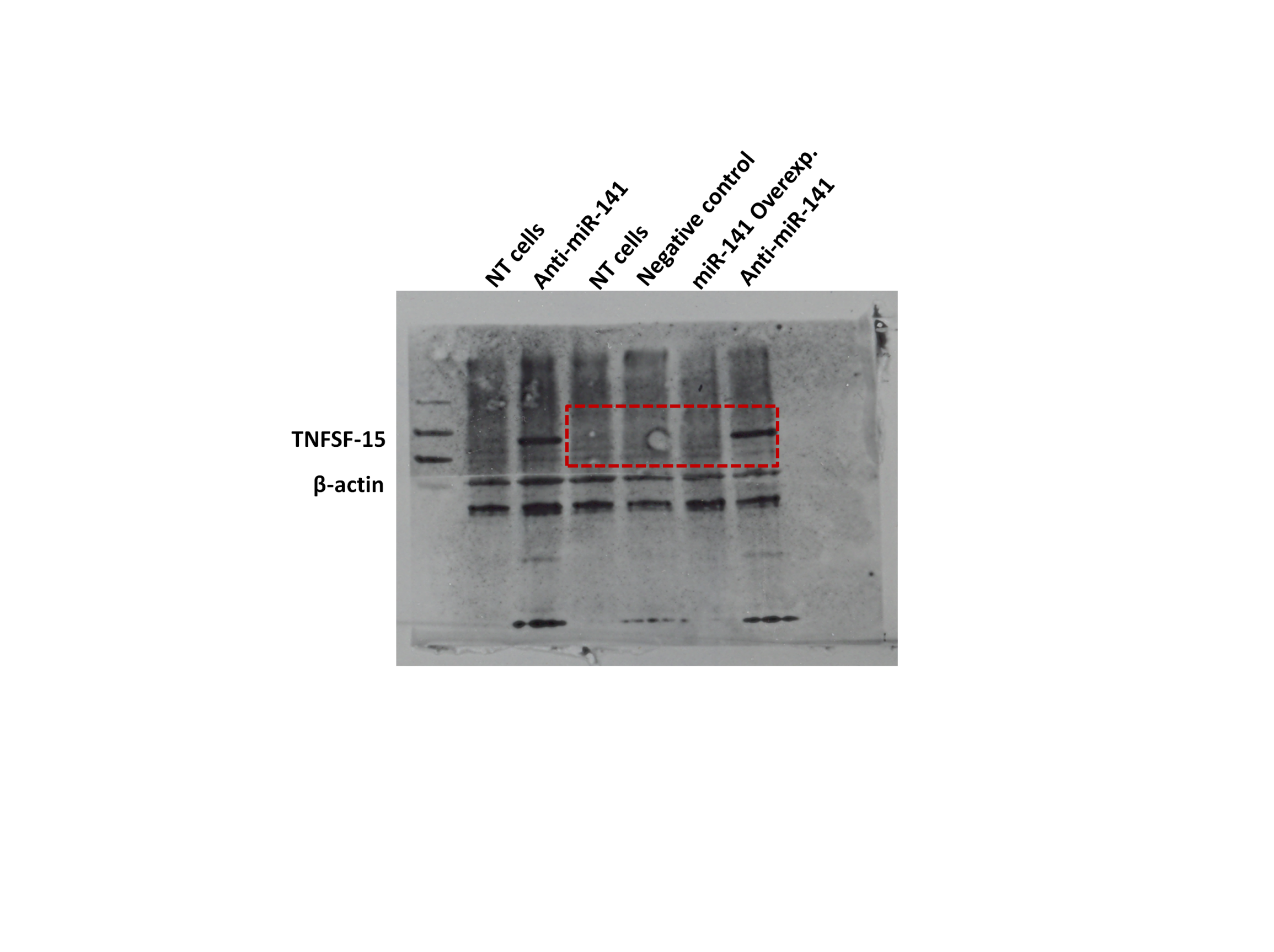


**Supp. Figure 3:** Original blots of TNFSF-15 immunoblotting membrane reveals the protein levels of TNFSF-15 in HepG2 cells that transfected with an inhibitor antagonist miR-141, or the miR-141 overexpression vector compared to non-transfected cells and other control transfected cells. β-actin was introduced as an internal control. β-actin was introduced as an internal control.


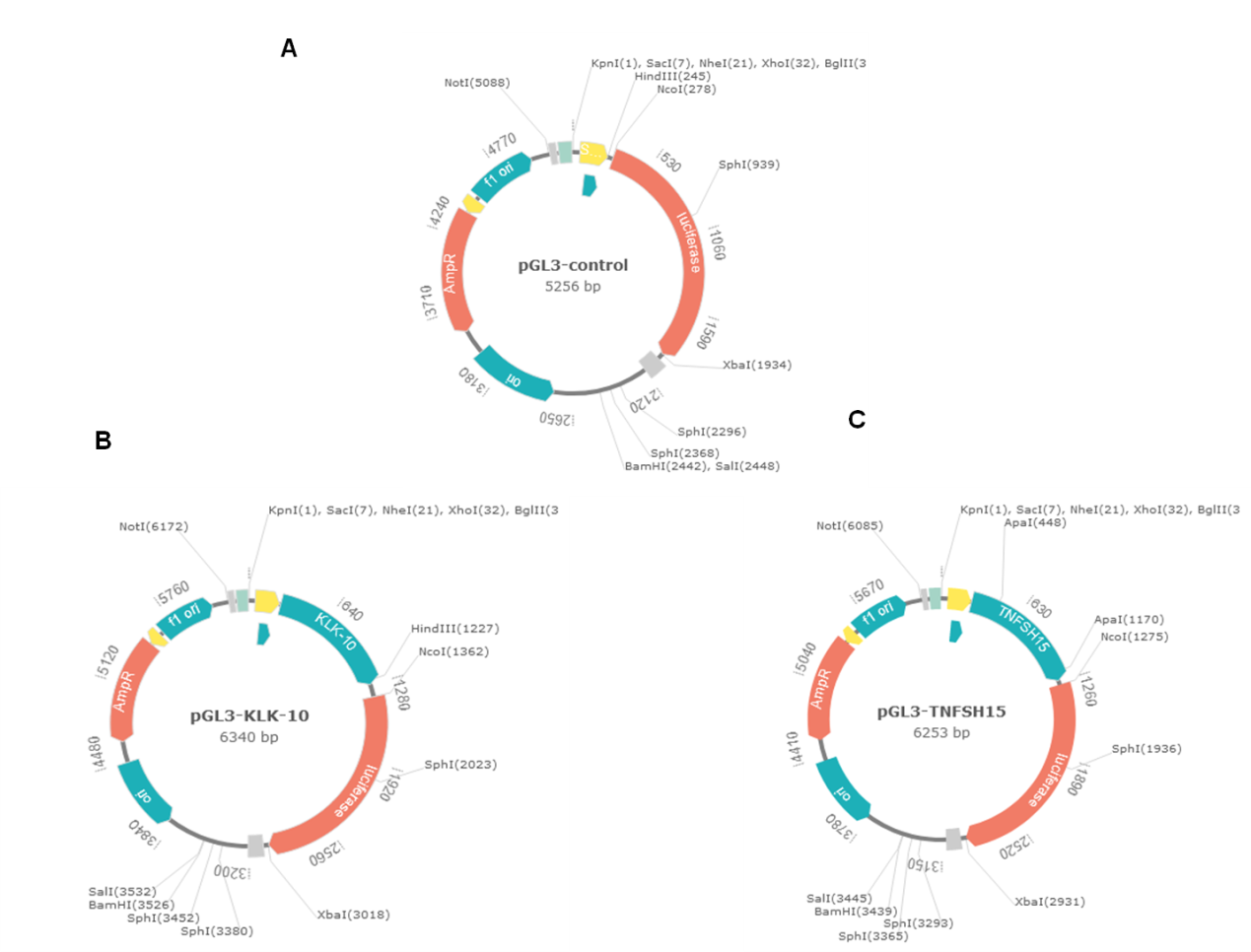


**Supp. Figure 4:** **Schematic representation of luciferase reporter constructs map** (A) pGL3-control vector, (B) pGL3-KLK10 construct, and (C) pGL3-TNFSF-15 showed the cloned seeding region in the coding sequences of each targeted gene inserted between SV40 promoter and luciferase reporter gene using online Molbiotools.


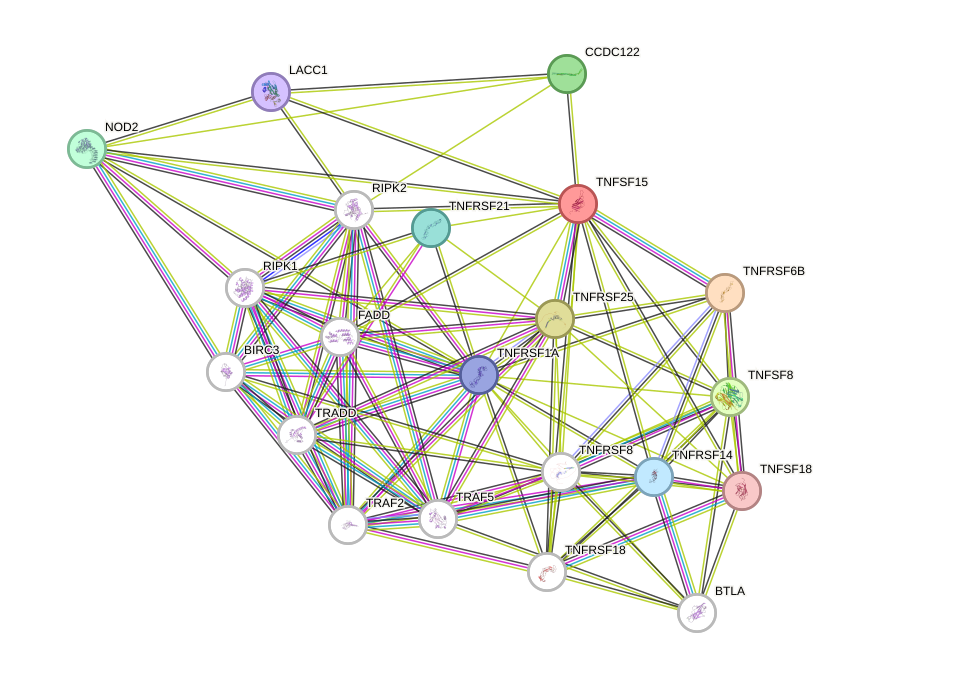


**Supp. Figure 5: TNFSF15 protein interaction network**

The TNFSF-15 is one of the shortlisted miRNAs targets which were selected from the microarray and then subjected to Search Tool for the Retrieval of Interacting Genes (STRING) 12 version database analyses. STRING database constructed a network model which allows displaying the interaction of all protein. This analysis is connected to both the KEGG and the GO databases to cluster the shortlisted genes as the input into various pathways and different biological processes. The confidence interval in this model is 0.4 which represents a broad range of predicted proteins to be interacted with TNFSF-15.


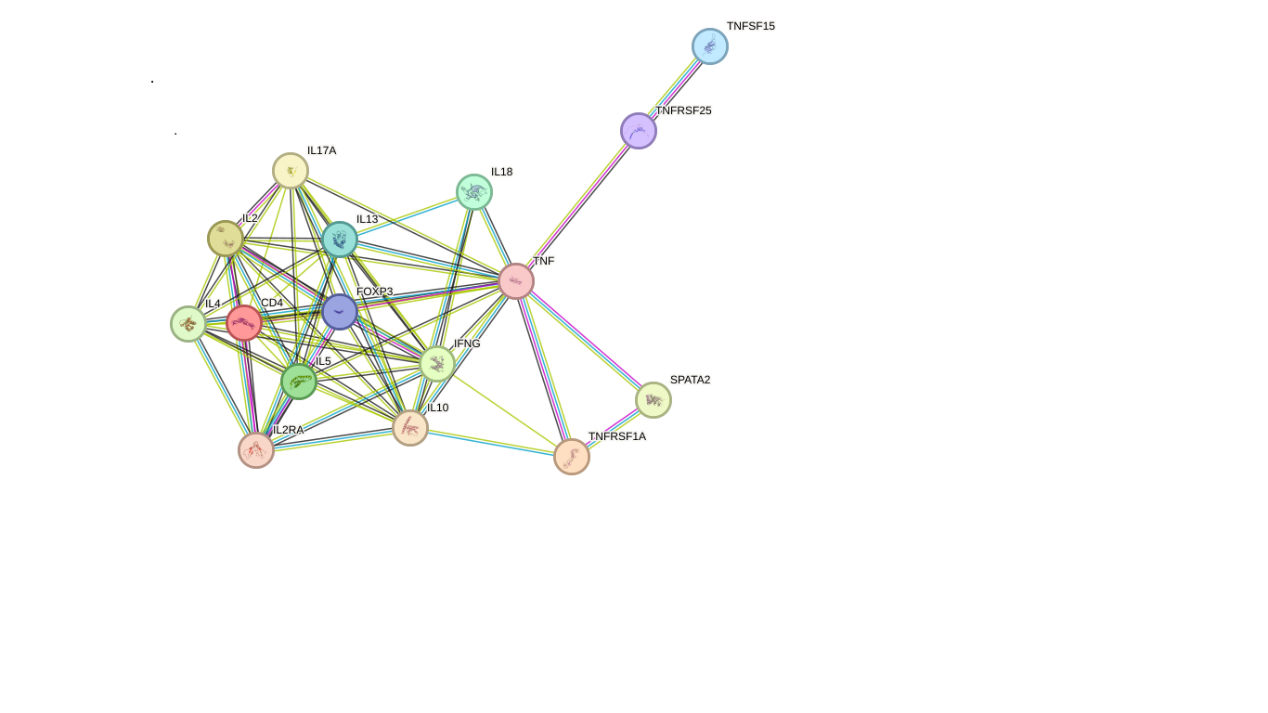


**Supp. Figure 6: TNFSF15 protein-protein Interaction with the highest confidence interval 0.90**

The TNFSF15 is one of the shortlisted miRNAs targets which were selected from the microarray and then subjected to Search Tool for the Retrieval of Interacting Genes (STRING) 12 version database analyses. STRING database constructed a network model which allows to display the interaction of all protein. This analysis is connected to both the KEGG and the GO databases to cluster the shortlisted genes as the input into various pathways and different biological processes. The confidence interval in this model is 0.9 which represents the most precise predicted specific range of predicted proteins to be interacted with TNFSF15.

**Supp. Table 1:** KEGG pathways and matched member genes function regulated by TNFSF-15

| **False discovery rate** | **Pathway description** | **Pathway/ ID** |
| --- | --- | --- |
| 0.000010 | Apoptosis- Multiple species | 04213 |
| 8.87e-11 | TNF- signaling pathway | 04668 |
| 7.56e-08 | NF-Kappa B signaling pathway | 04064 |
| 2.37e-05 | Reg-like receptor signaling pathway | 04622 |
| 1.83e-08 | necroptosis | 04217 |
| 2.41e-07 | Apoptosis | 04210 |
| 5.61e-05 | IL-17 signaling pathway | 04657 |
| 0.00095 | Adipocytokine signaling pathway | 04920 |
| 4.12e-08 | NOD-like receptor signaling pathway | 04621 |
| 9.81e-08 | Salmonella infection | 05132 |
| 0.0022 | Small cell lung cancer | 05222 |
| 1.24e-09 | Cytokine-Cytokine receptor interaction | 04060 |
| 1.80e05 | Hepatitis C | 05160 |
| 2.11e-05 | Tuberculosis | 05152 |
| 2.63e-06 | Human immunodeficiency virus 1 infection | 05170 |
| 0.0341 | Platinum drug resistance | 01524 |
| 3.44e-06 | Human cytomegalo virus infection | 05163 |
| 3.44e-06 | Shigellosis | 05131 |
| 3.34e-05 | Pathogenic E. coli infection | 05130 |
| 3.55e-05 | Epstein-Bvirus infection | 05169 |
| 0.0040 | Sphingolipid signaling pathway | 04071 |
| 0.00079 | Kaposi Sarcoma associated herpes virus infection | 05167 |
| 0.0102 | Influenza A | 05164 |
| 0.0136 | Viral carcinogenesis | 05203 |
| 1.37e-05 | Herpes simplex virus | 05168 |
| 0.0424 | MAPK signaling pathway | 04010 |
| 0.0267 | Pathways in cancer | 05200 |
